# Supplementary material for: SRPK1 acetylation modulates alternative splicing to regulate cisplatin resistance in breast cancer cells
Source: Commun Biol. 2020 May 27;3:268. doi: 10.1038/s42003-020-0983-4 (PMC7253463; doi:10.1038/s42003-020-0983-4)

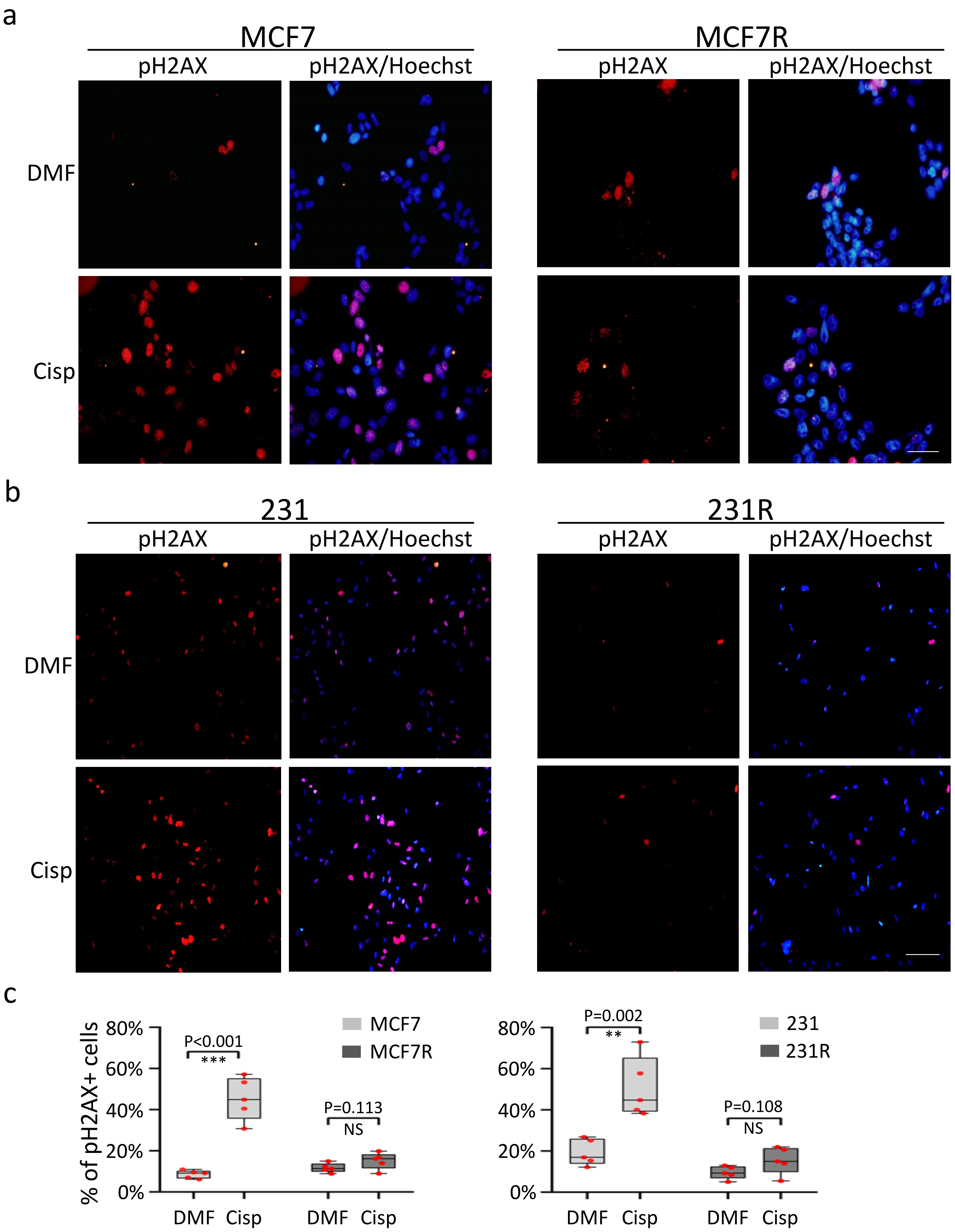

Supplementary Figure 1

Supplementary Figure 1. Verification of cisplatin-resistant breast cancer cells. MCF7/MCF7R (**a**) and 231/231R cells (**b**) were treated with DMF or 10 µM cisplatin for 5 days. The extent of DNA damage was assessed by immunostaining for pH2AX. The nuclei were counterstained with Hoechst 33342, and pH2AX-positive cells were counted (**c**). Scale bar: 100 µm. Boxplot: n=5; ***p*<0.01, ****p*<0.001 by Student's *t*-test. NS: no significance.

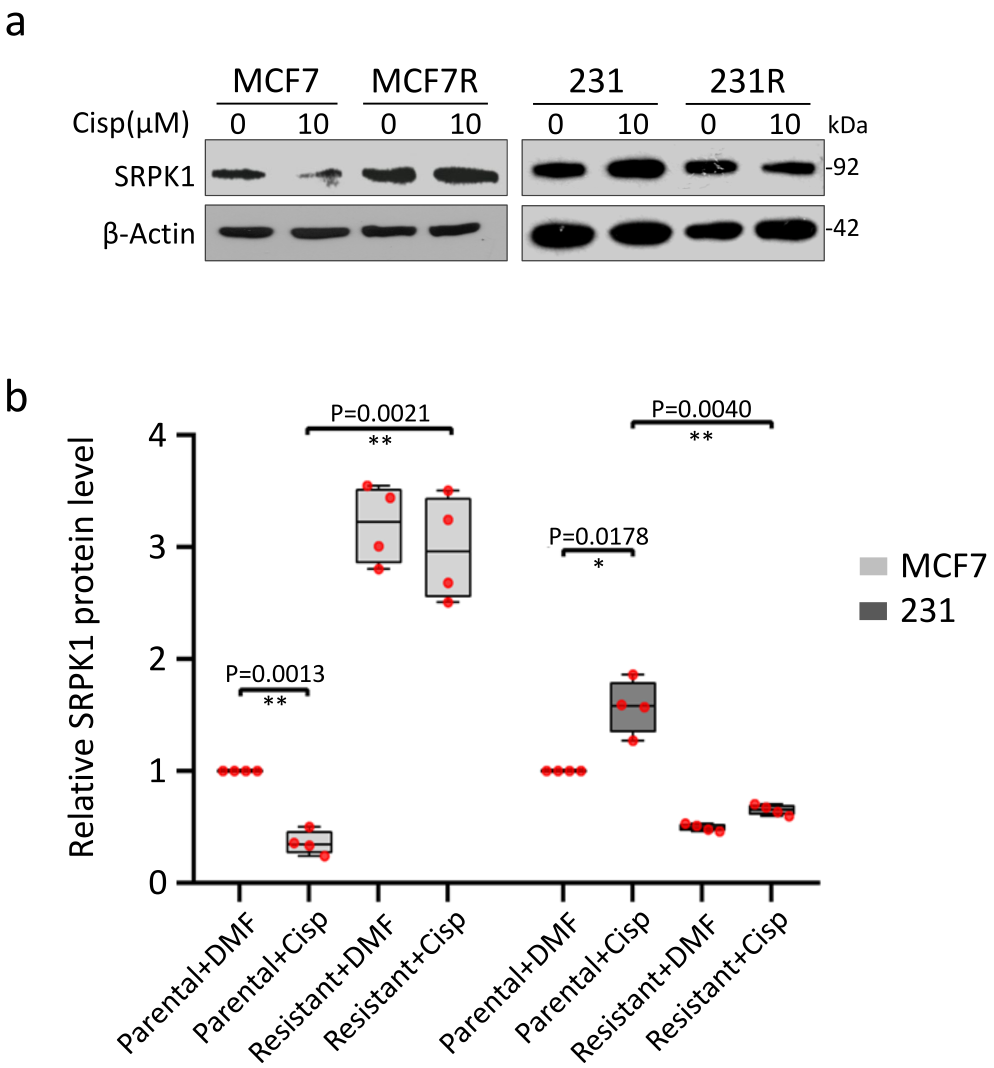

Supplementary Figure 2. The effect of cisplatin on SRPK1 expression. (**a**) The indicated cells were treated with DMF or 10 μM cisplatin for 5 days. Immunoblotting was then performed with the SRPK1 antibody. The blots are representative of four experiments with similar results. (**b**) Quantification of SRPK1 expression in relative to β-Actin. Boxplot: n=4; *$p<0.05$, **$p<0.01$ by Student's *t*-test.

Supplementary Figure 2

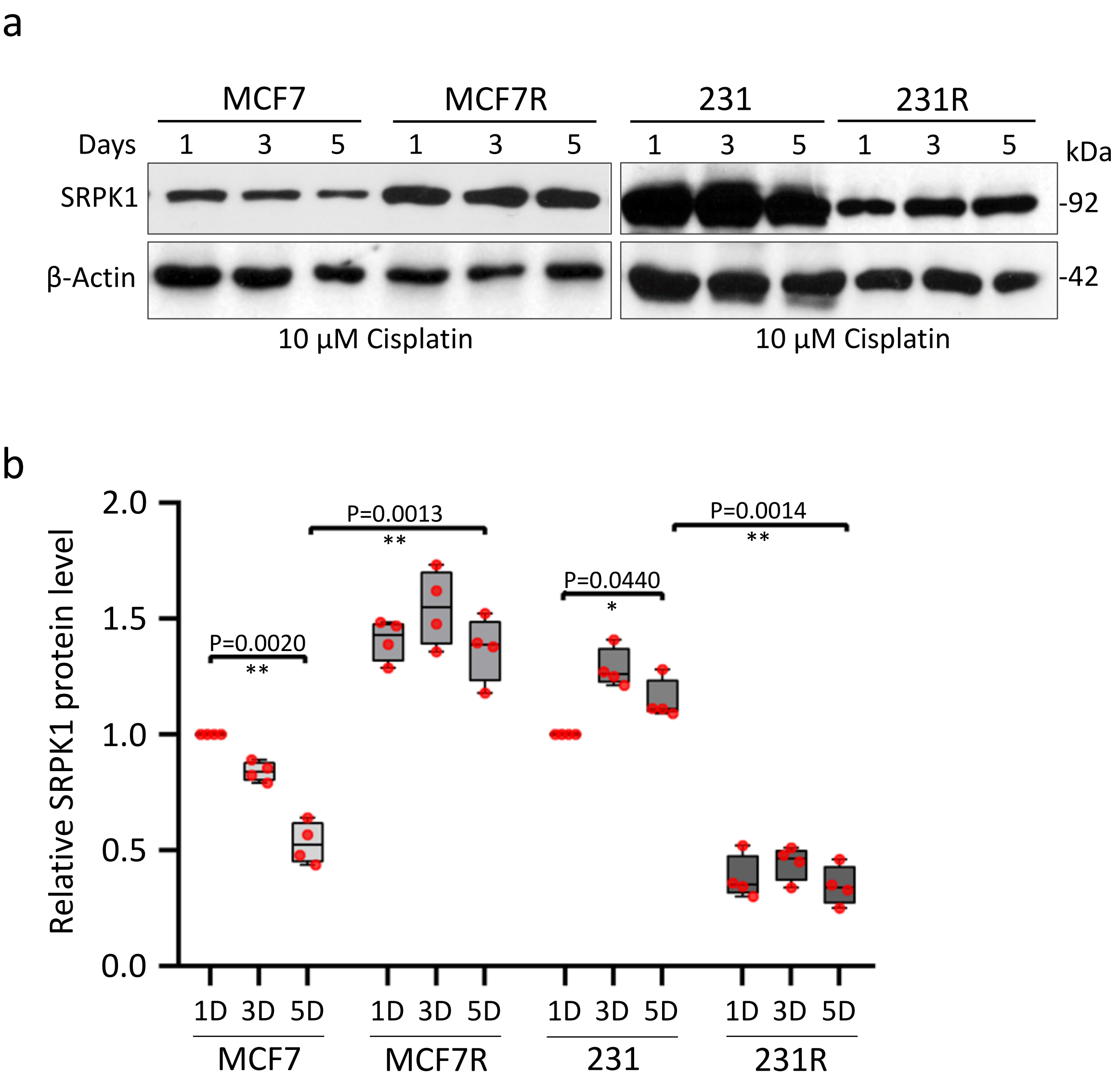

Supplementary Figure 3. The time-course effect of cisplatin on SRPK1 expression. (**a**) The indicated cells were treated with 10 μM cisplatin for 1 day, 3 days and 5 days. The SRPK1 protein level was determined by immunoblotting. The blots are representative of four experiments with similar results. (**b**) Quantification of SRPK1 expression in relative to β-Actin. Boxplot: n=4; **p*<0.05, ***p*<0.01 by Student's *t*-test.

a

Acetylation site	Sequence	SRPK1 +Tip60	SRPK1 +Tip60-mut
215K	IIHTDI**<u>K</u>**PE	+	–
258K	SGAPPPSGSAVSTAPQP**<u>K</u>**PADKMSK	+	–
265K	SGAPPPSGSAVSTAPQPKPADKMS**<u>K</u>**	+	–
301K	MQEIEEMEKESGPGQ**<u>K</u>**	+	–
318K	QEESESPVERPL**<u>K</u>**ENPPNK	+	–

b

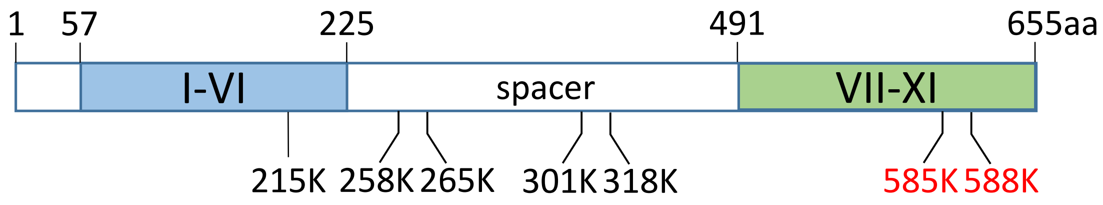

Supplementary Figure 4. Potential sites of acetylation in SRPK1. (**a**) SRPK1 was co-expressed with Tip60 or the HAT-deficient mutant, Q377E/G380E, in 293T cells. The acetylation of SRPK1 was analysed by mass spectrometry. The acetylated residues are in bold and underlined. (**b**) The positions of potential acetylated lysine residues in SRPK1. The sites in red are reported by Choudhary et al. 2009 [27].

Supplementary Figure 4

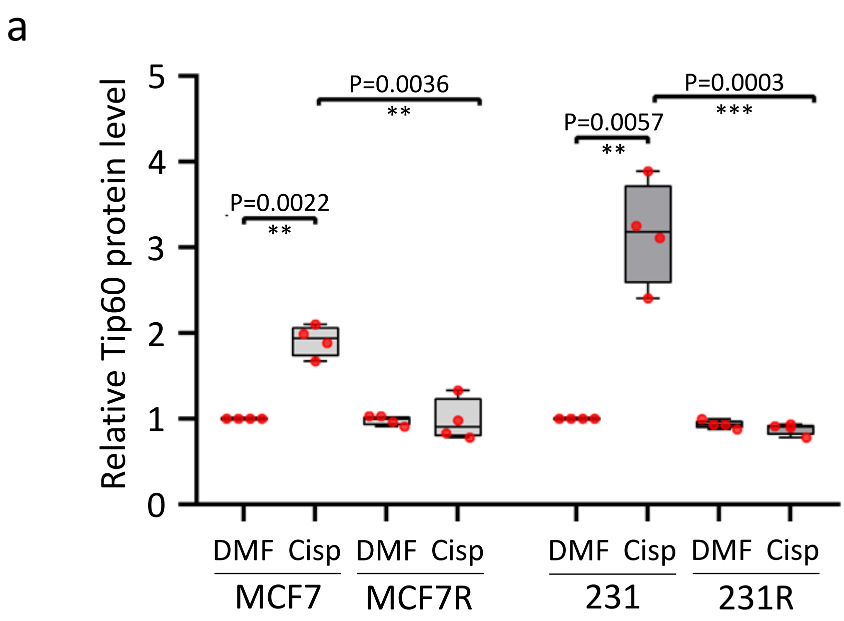

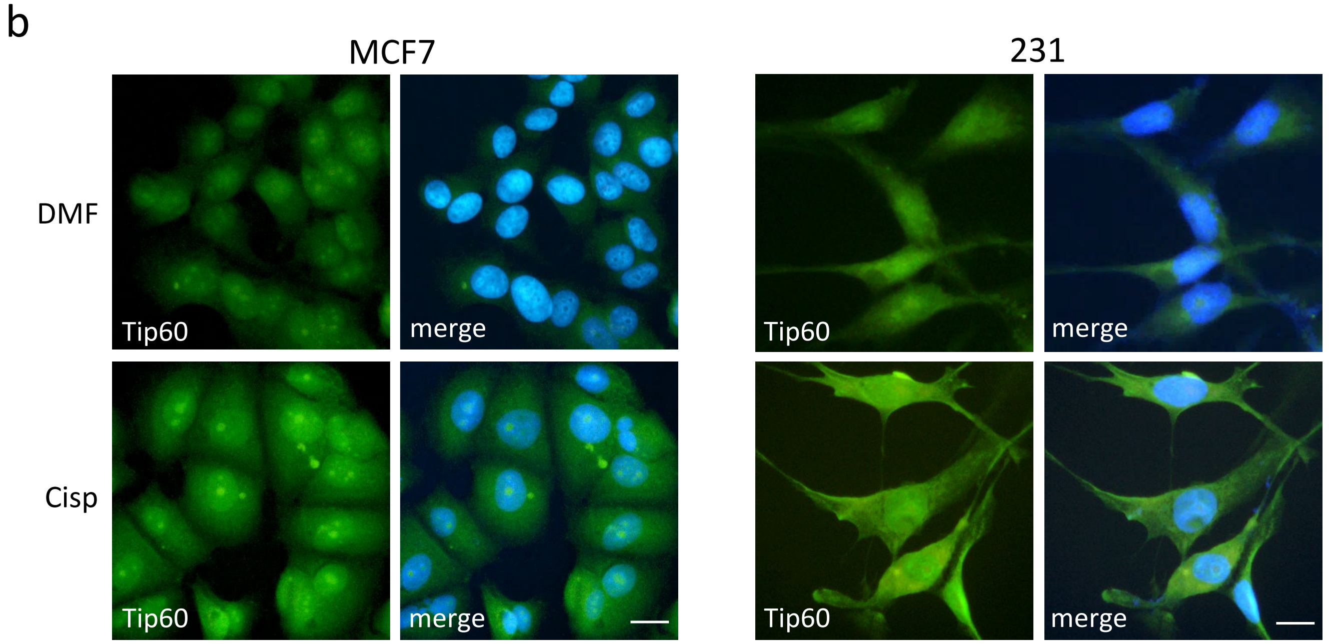

Supplementary Figure 5. Tip60 was increased by cisplatin treatment in the parental cells. **(a)** Quantification of Tip60 protein level in relative to β-Actin in the indicated cells. Boxplot: n=4; $**p<0.01$, $***p<0.001$ by Student's *t*-test. (**b**) MCF7 and 231 cells were treated with DMF or 10 μM cisplatin for 5 days. Immunofluorescence was then performed to detect the expression and subcellular localization of Tip60. The nuclei were counterstained with Hoechst 33342. Scale bar: 20 μm. The images are representative of three experiments with similar results.

Supplementary Figure 5

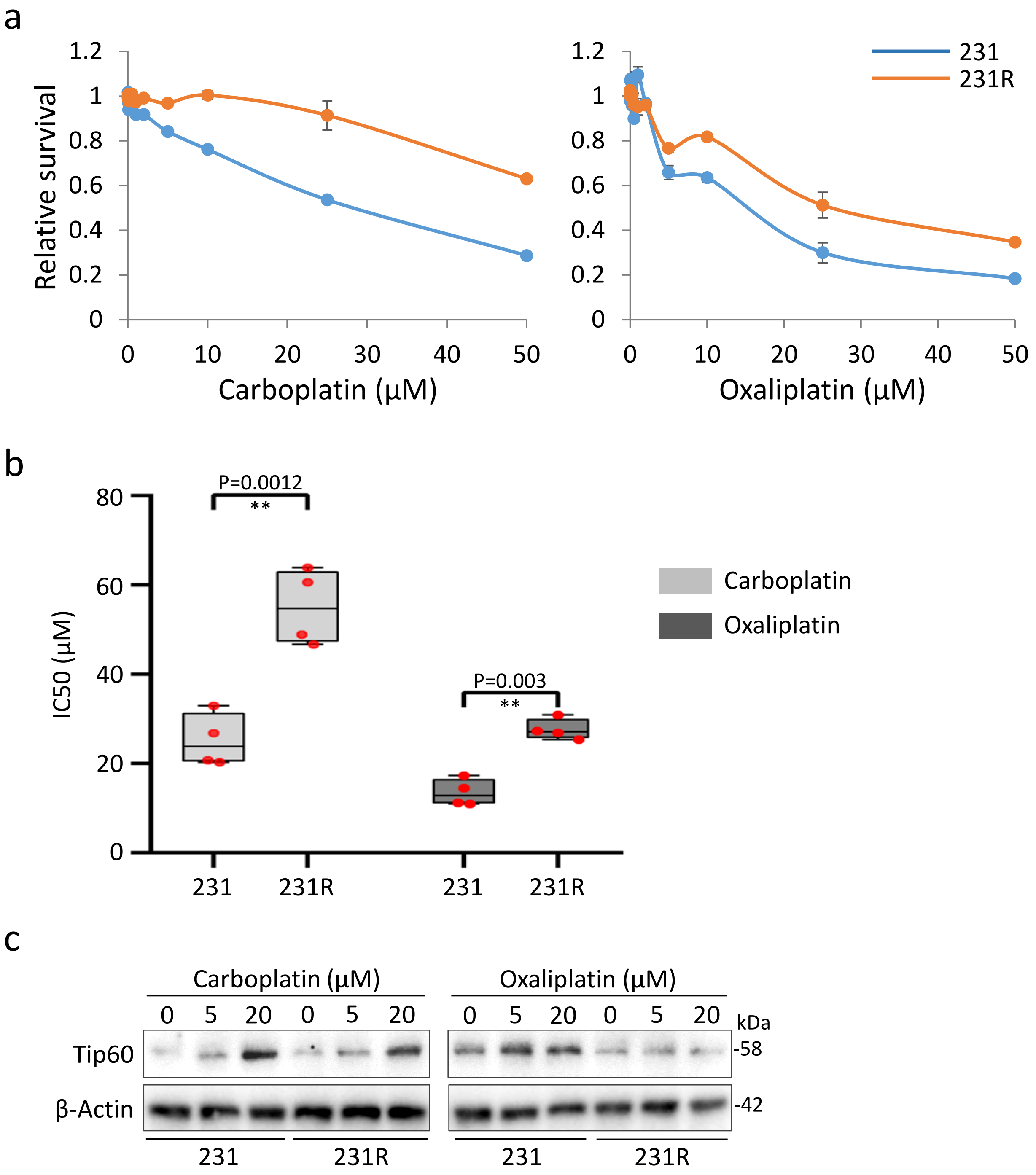

Supplementary Figure 6

Supplementary Figure 6. The effect of carboplatin and oxaliplatin on Tip60 expression. (**a**) 231 and 231R cells were treated with carboplatin or oxaliplatin. The cell survival was then assessed by the MTS viability assay. The reading was normalized to DMSO-treated cells. Data points: mean ± SD; n=3. (**b**) The IC50 of carboplatin and oxaliplatin for the indicated cell lines was calculated using the Hill equation. Boxplot: n=4; $**p<0.01$ by Student's *t*-test. (**c**) The cells were treated with the indicated doses of carboplatin and oxaliplatin for 2 days and the protein level of Tip60 was checked by immunoblotting. The blots are representative of three experiments with similar results.

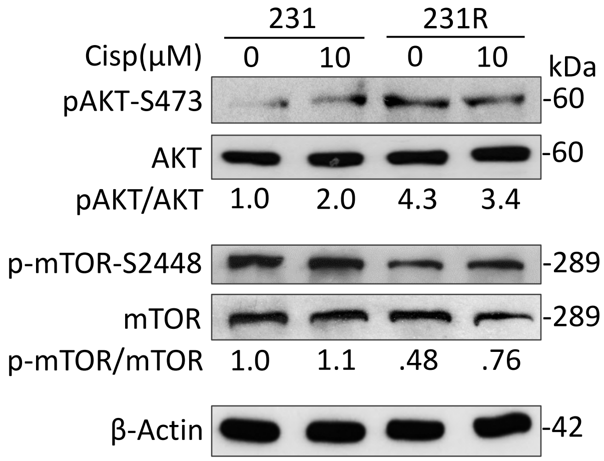

Supplementary Figure 7. The activation status of AKT/mTOR in 231 and 231R cells. The indicated cells were treated with DMF or 10 µM cisplatin for 5 days. Then the cells were subject to immunoblotting to detect the activation of AKT and mTOR with indicated antibodies. The decimals below the gel strips denote the relative abundance of phosphorylated AKT (pAKT) or phosphorylated mTOR (p-mTOR) against the respective total protein. The blots are representative of three experiments with similar results.

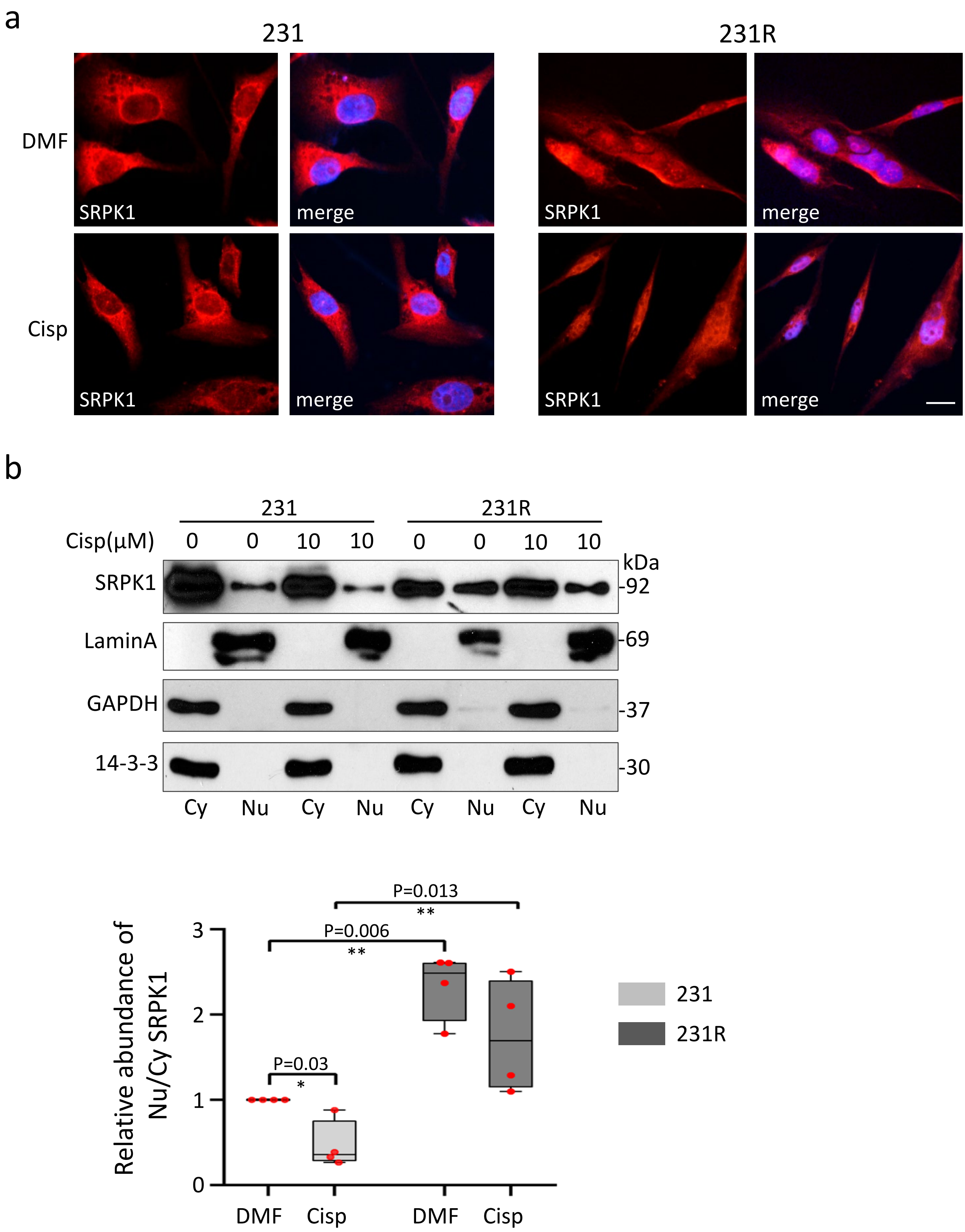

Supplementary Figure 8

Supplementary Figure 8. Acetylation could affect the subcellular localization of SRPK1. (**a**) 231 and 231R cells were treated with cisplatin for 5 days and immunostained for SRPK1. The nuclei were counterstained with Hoechst 33342. Scale bar: 20 µm. The images are representative of three experiments with similar results. (**b**) Cisplatin-treated 231 and 231R cells were fractionated into cytoplasmic (Cy) and nuclear (Nu) portions. The relative abundance of SRPK1 in each fraction was assessed by immunoblotting. LaminA was used as the marker for the nuclear fraction; GAPDH and 14-3-3β for the cytoplasmic fraction. The blots are representative of four experiments with similar results. The Nu/Cy ratio was normalized against the "231+DMF" group. Boxplot: n=4; *p<0.05, **p<0.01 by Student's *t*-test.

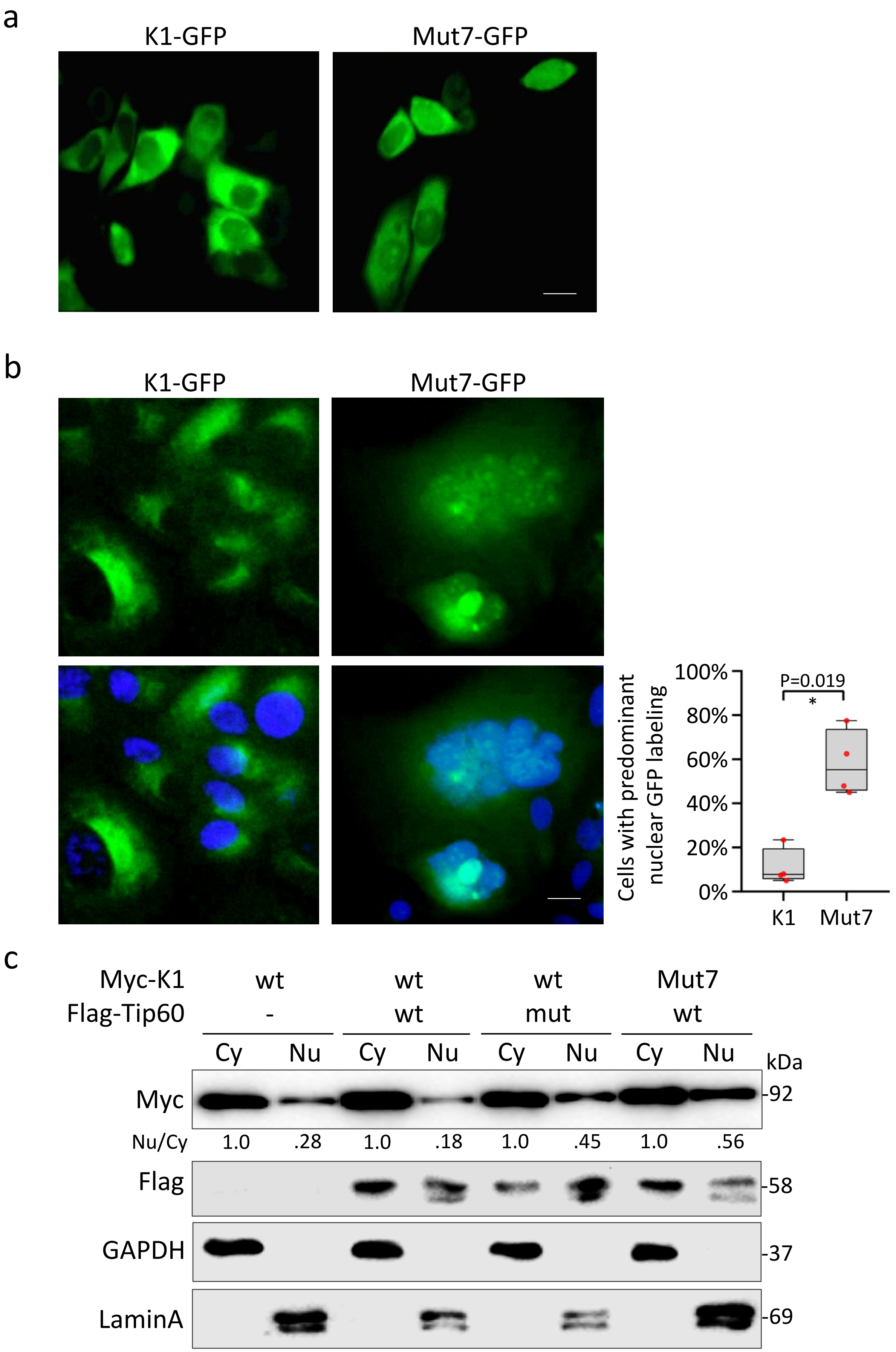

Supplementary Figure 9

Supplementary Figure 9. Acetylation could affect the subcellular localization of SRPK1. (**a**) HeLa were transfected with GFP-tagged SRPK1 (K1-GFP) or Mut7 (Mut7-GFP). The subcellular localization of GFP signals was examined by the live cell imaging. (**b**) MCF-7 cells were similarly transfected as HeLa in (**a**). The cells were then fixed and the nuclei counterstained with Hoechst 33342. Scale bar: 20 µm. Boxplot: n=4; *p<0.05 by Student's *t*-test. (**c**) HeLa cells were transfected with the indicated constructs and subjected to cytoplasmic (Cy) and nuclear (Nu) fractionation. The lysates were then analysed by immunoblotting to study the subcellular localization of overexpressed Myc-tagged SRPK1 (Myc-K1). GAPDH and LaminA were used as markers for the cytoplasmic and nuclear fractions, respectively. The decimals below the gel strip of anti-Myc denote the relative abundance of nuclear vs. cytoplasmic SRPK1. wt: wild-type; mut: HAT-deficient Tip60; Mut7: acetylation-deficient SRPK1. For immunofluorescence images in panels (**a, b**) and Western blots in (**c**), they are representative of four and three experiments with similar results, respectively.

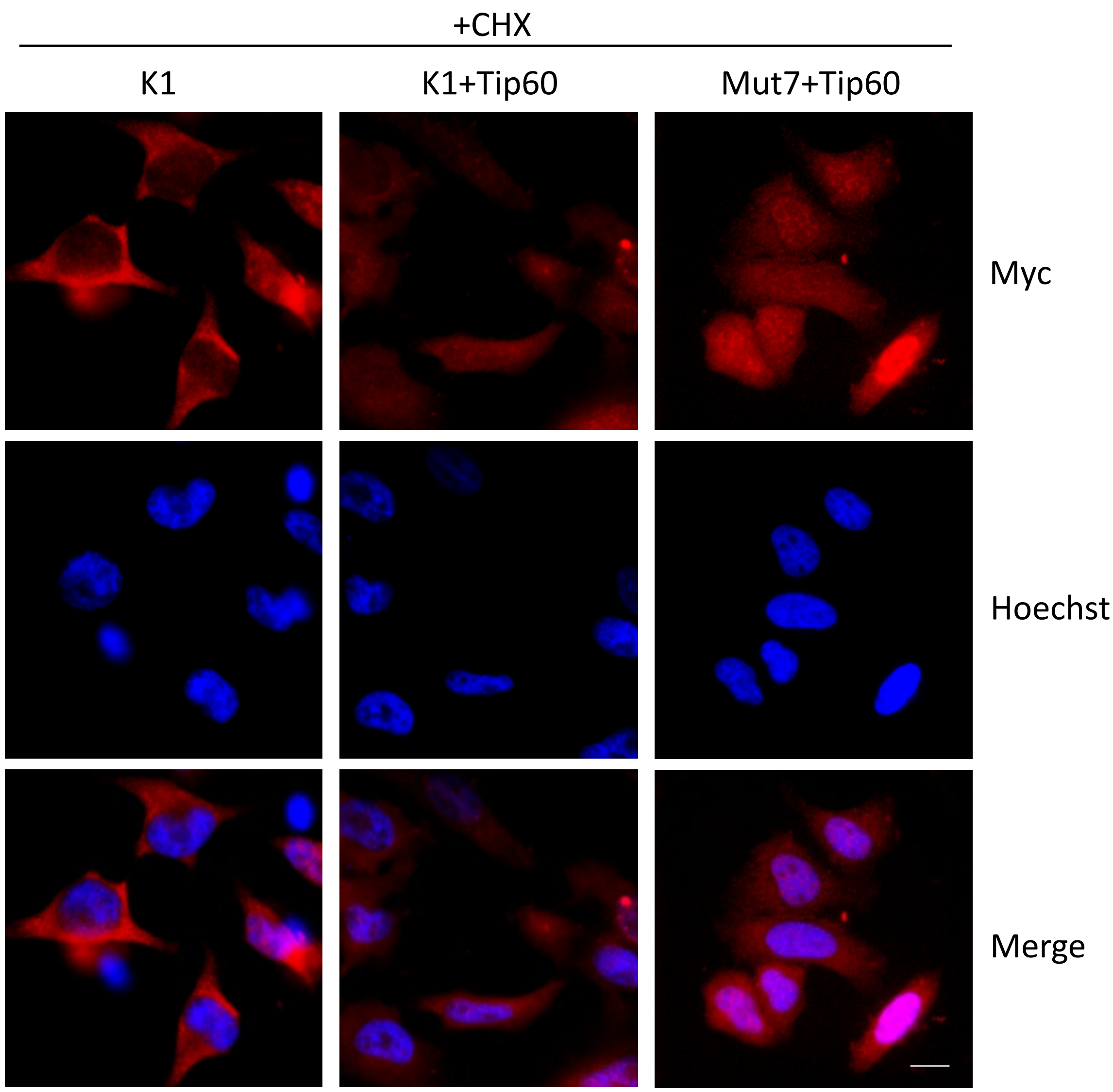

Supplementary Figure 10. The protein stability of SRPK1 was affected by acetylation. 293T cells were transfected with Myc-tagged SRPK1 (K1) or Mut7 and Tip60 as indicated. The cells were then immunostained with the c-Myc antibody. The nuclei were counterstained with Hoechst 33342. Scale bar: 5 μm. The images are representative of three experiments with similar results.

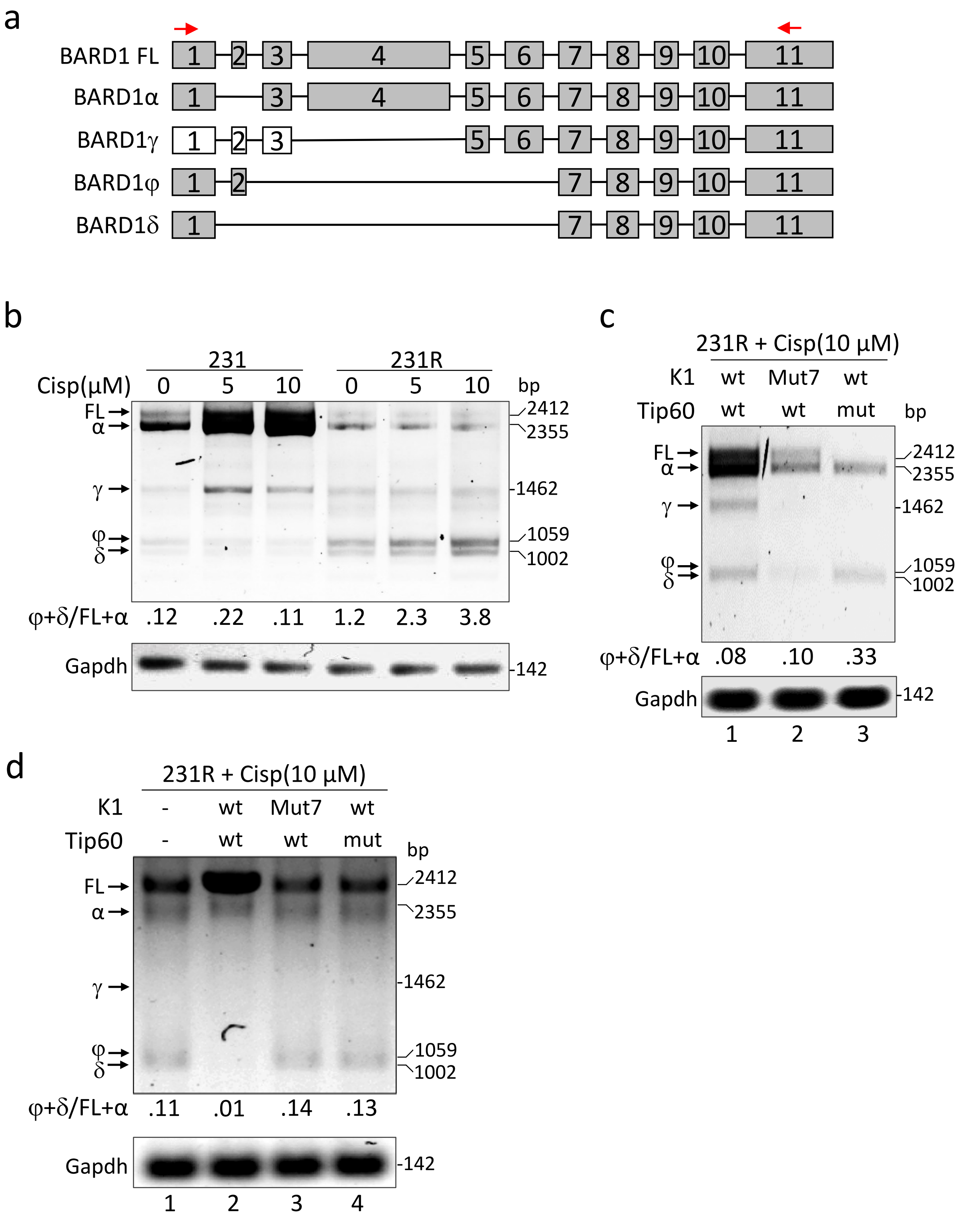

Supplementary Figure 11

Supplementary Figure 11. Alternative splicing of *BARD1* in the parental and cisplatin-resistant cells. (**a**) Schematic structures of *BARD1* and its splice variants. The red arrows indicate the primers used for PCR. The grey boxes indicate coding exons and white boxes noncoding exons. (**b**) 231 and 231R cells were treated with cisplatin of indicated concentrations for 5 days. The transcript levels of *BARD1* splice variants were checked by RT-PCR. (**c, d**) 231R cells were transfected with SRPK1 and Tip60 constructs as indicated and treated with cisplatin. The splicing of *BARD1* was then examined by RT-PCR. wt: wild-type; Mut7: acetylation-deficient SRPK1; mut: HAT-deficient Tip60. The decimals below the gel strips (**b-d**) denote the relative abundance of oncogenic variants versus tumour suppressive variants. The RT-PCR gel images in (**b-d**) are representative of three experiments with similar results.

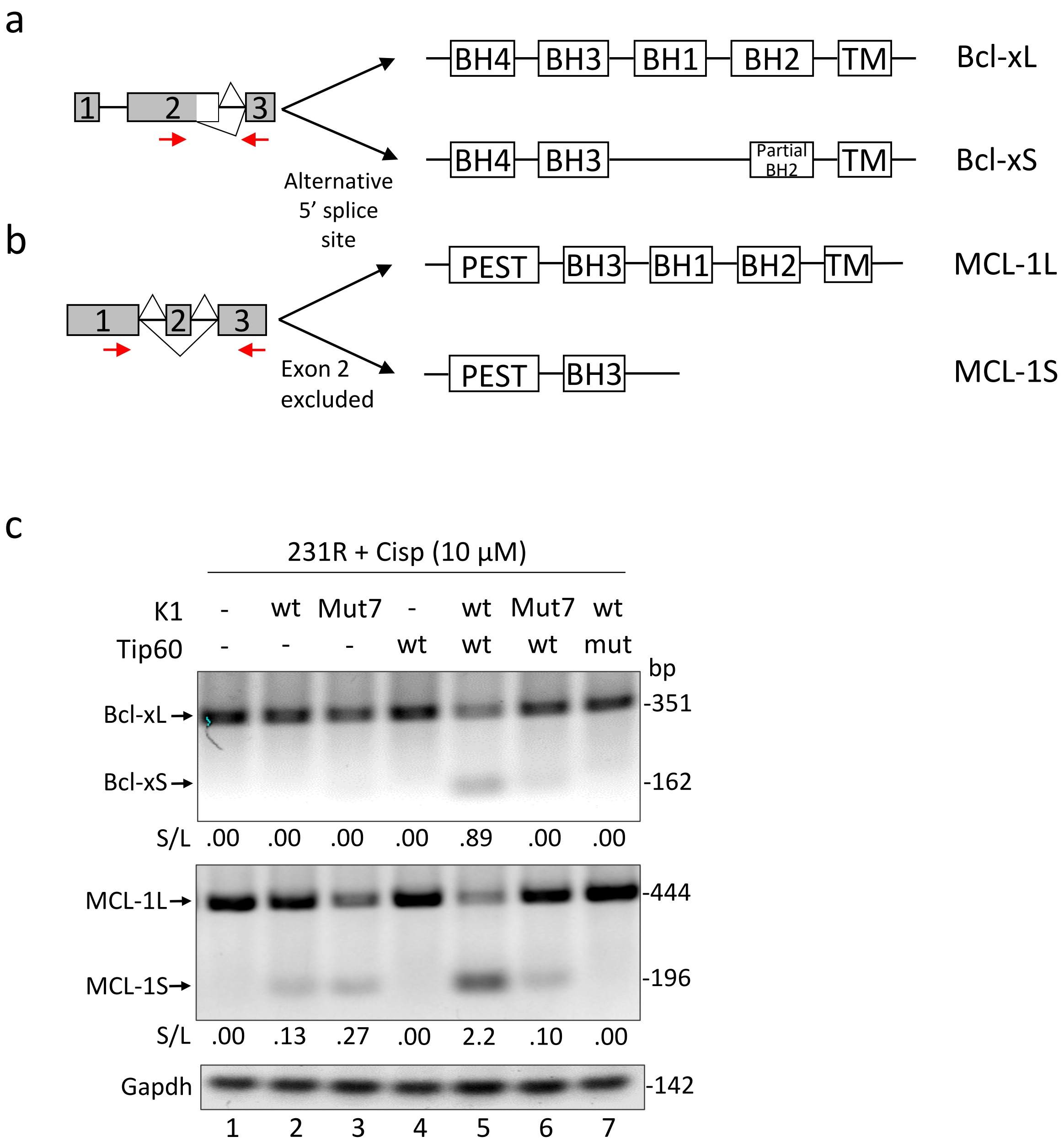

Supplementary Figure 12. Alternative splicing of *BCL2L1* and *MCL-1* could be affected by SRPK1 acetylation. (**a, b**) Schematic structures of *BCL2L1*, *MCL-1* and their splice variants. The red arrows indicate the primers used for PCR. (**c**) 231R cells were transfected with SRPK1 and Tip60 constructs as indicated, and treated with cisplatin for 5 days. The levels of alternatively spliced variants of *BCL2L1*, *MCL-1* and *BARD1* were checked by RT-PCR. The decimals below the gel strips denote the relative abundance of pro-apoptotic versus anti-apoptotic variants. The RT-PCR gel images in (**c**) are representative of three experiments with similar results.

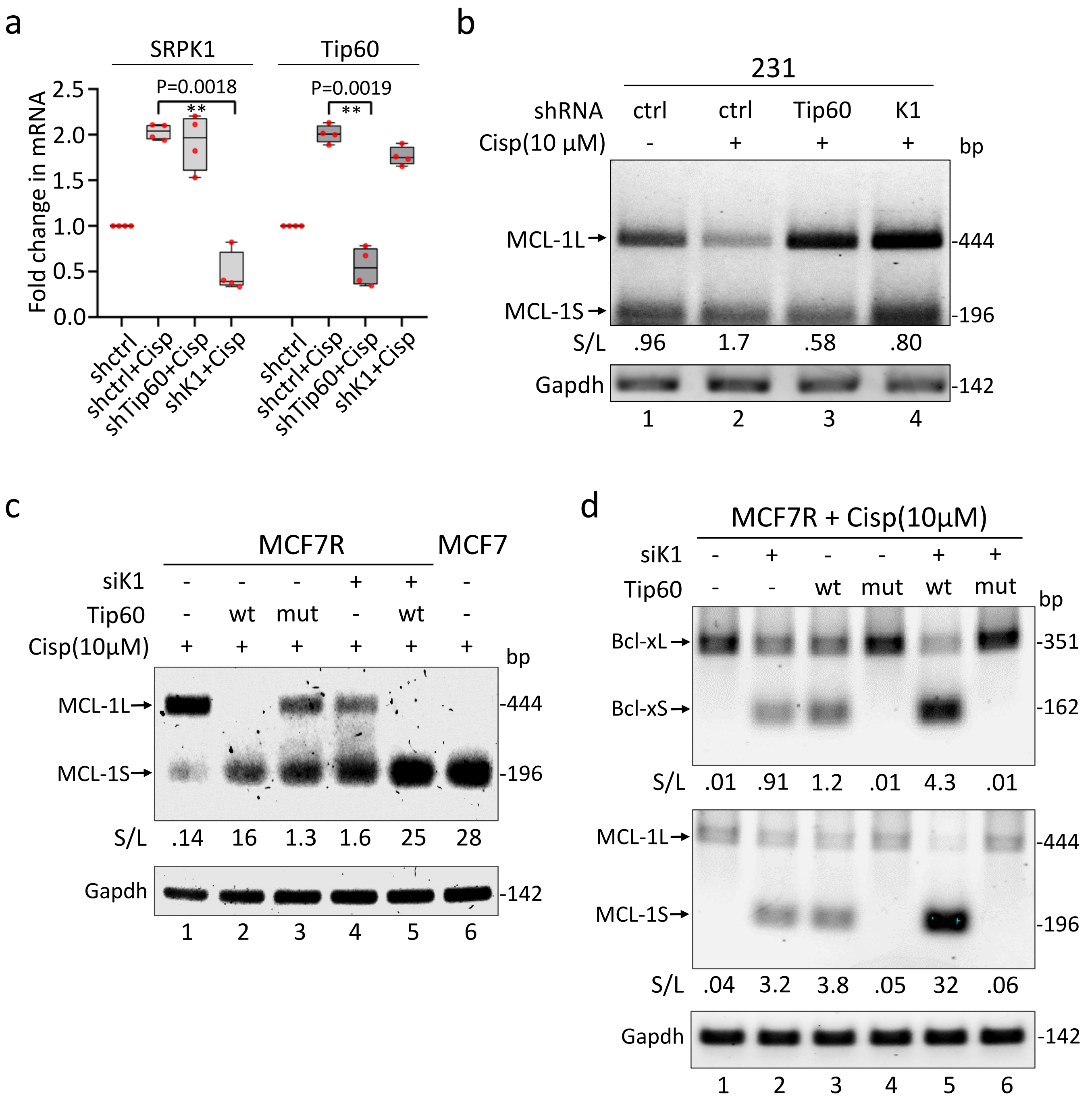

Supplementary Figure 13

Supplementary Figure 13. Alternative splicing of *BCL2L1* and *MCL-1* could be affected by SRPK1 acetylation. (**a**) 231 cells were transfected with the shRNAs targeting Tip60 or SRPK1 and treated with cisplatin. The knockdown efficiency was then determined by qPCR. Boxplot: n=4; **$p<0.01$ as determined by Student's *t*-test. (**b**) 231 cells were transfected with shRNAs targeting Tip60 or SRPK1 and treated with cisplatin. The splicing of *MCL-1* was checked by RT-PCR. (**c, d**) MCF7R cell were transfected with the siRNA pool targeting SRPK1 (siK1) and Tip60 constructs as indicated, and then treated with cisplatin. The splicing of *MCL-1* and *BCL2L1* was evaluated by RT-PCR. MCF7 was included as a negative control. The decimals below the gel strips in (**c, d**) denote the relative abundance of short (S) versus long (L) variants. wt: wild-type Tip60; mut: HAT-deficient Tip60. The RT-PCR gel images in (**b-d**) are representative of three experiments with similar results.

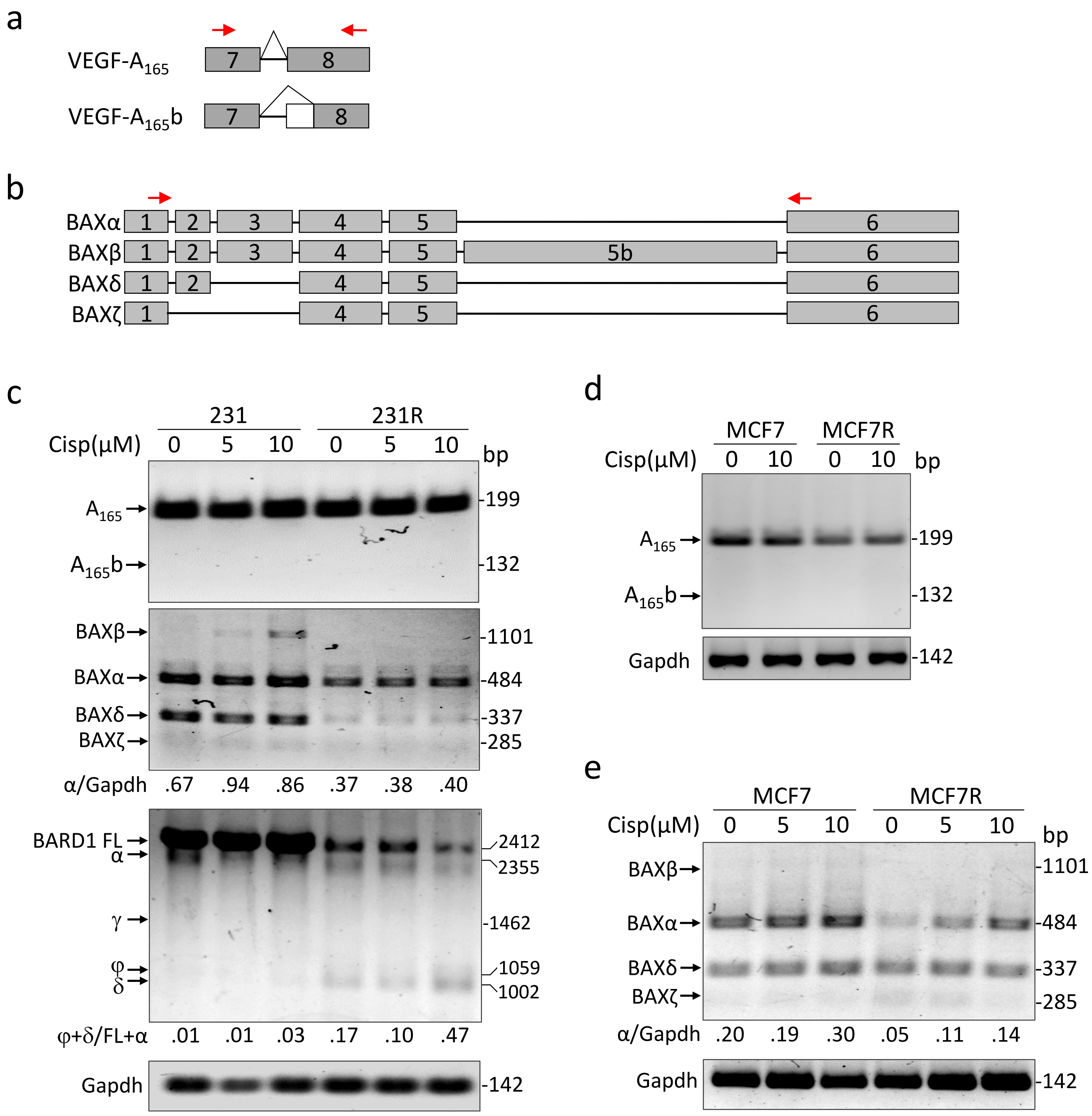

Supplementary Figure 14

Supplementary Figure 14. Alternative splicing of *VEGF-A*$_{165}$ and *BAX* in the parental and cisplatin-resistant cells. (**a, b**) Schematic structures of *VEGF-A*$_{165}$ and *BAX*, and the respective splice variants. The red arrows indicate the primers used for PCR. The grey boxes indicate coding exons and white boxes noncoding stretch. (**c**) 231 and 231R cells were treated with cisplatin of indicated concentrations. The alternative splicing of *VEGF-A*$_{165}$ and *BAX*, as well as *BARD1* was examined by RT-PCR. (**d, e**) MCF7 and MCF7R cells were treated with cisplatin of indicated concentrations. The alternative splicing of *VEGF-A*$_{165}$ (**d**) and *BAX* (**e**) was examined by RT-PCR. The decimals below the gel strips in (**c, e**) denote the relative abundance of indicated variants. The RT-PCR gel images in (**c-e**) are representative of three experiments with similar results.

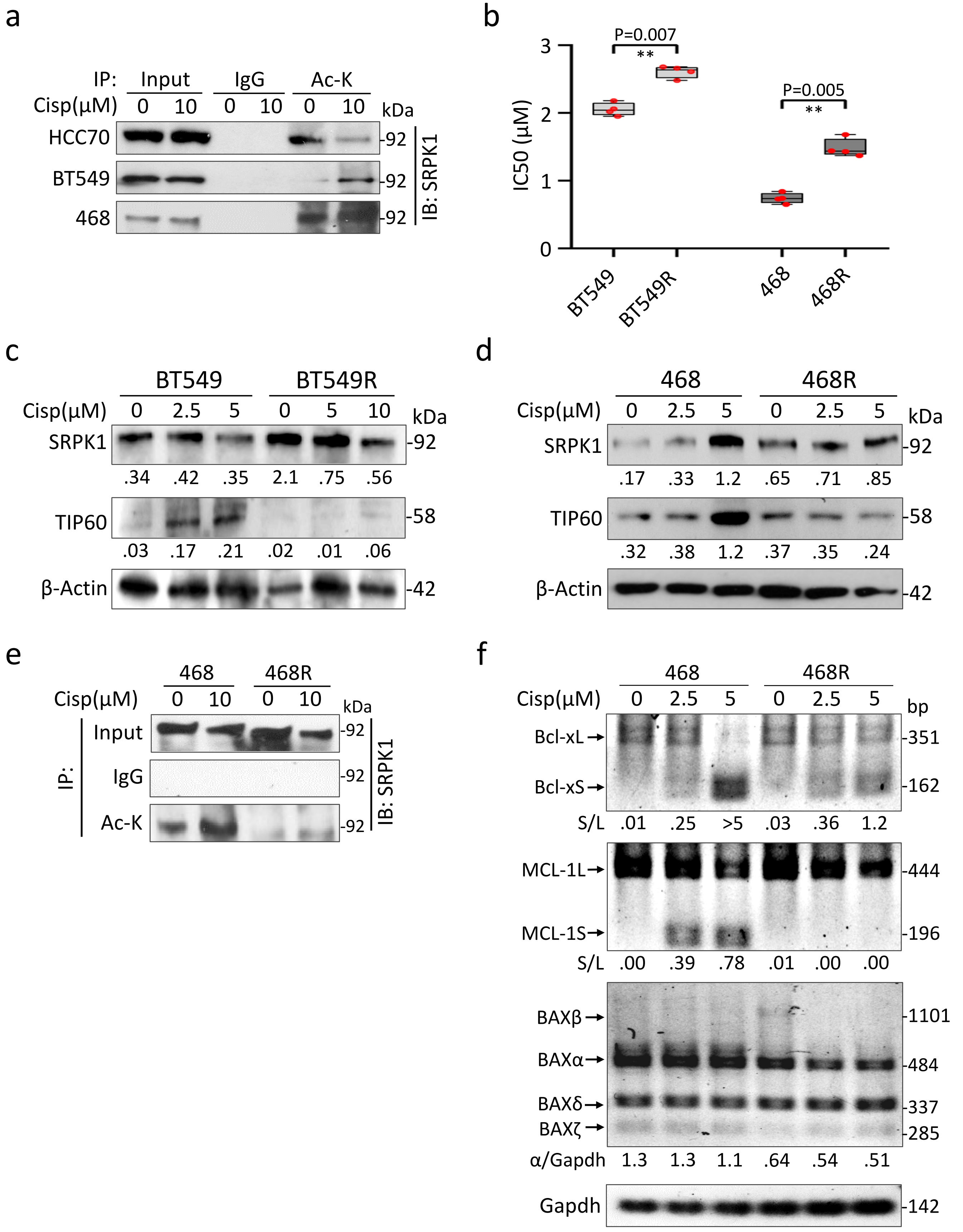

Supplementary Figure 15

Supplementary Figure 15. Acetylation of SRPK1 could be observed in multiple TNBC cells. (**a**) HCC70, BT549 and 468 cells were treated with DMF or cisplatin for 2 days, then the acetylation of SRPK1 was assessed by immunoprecipitation with the Ac-K antibody. (**b**) The cell survival of parental and resistant BT549 or 468 cells was assessed by the MTS viability assay. Boxplot: n=4; $^{**}p<0.01$ by Student's *t*-test. (**c, d**) BT549/BT549R (**c**) and 468/468R cells (**d**) were treated with DMF or cisplatin for 5 days. The protein levels of Tip60 and SRPK1 were determined by immunoblotting. The decimals below the gel strips denote the protein abundance in relative to β-Actin. (**e**) 468 and 468R cells were treated with DMF or cisplatin for 2 days. SRPK1 acetylation was then determined by using the Ac-K antibody in immunoprecipitation. (**f**) The splicing of *BCL2L1*, *MCL-1* and *BAX* in cisplatin-treated 468 and 468R cells was assessed by RT-PCR. The decimals below the gel strips denote the relative abundance of indicated variants. For Western blots in (**a, c-e**) and RT-PCR gel images in (**f**), they are representative of three experiments with similar results.

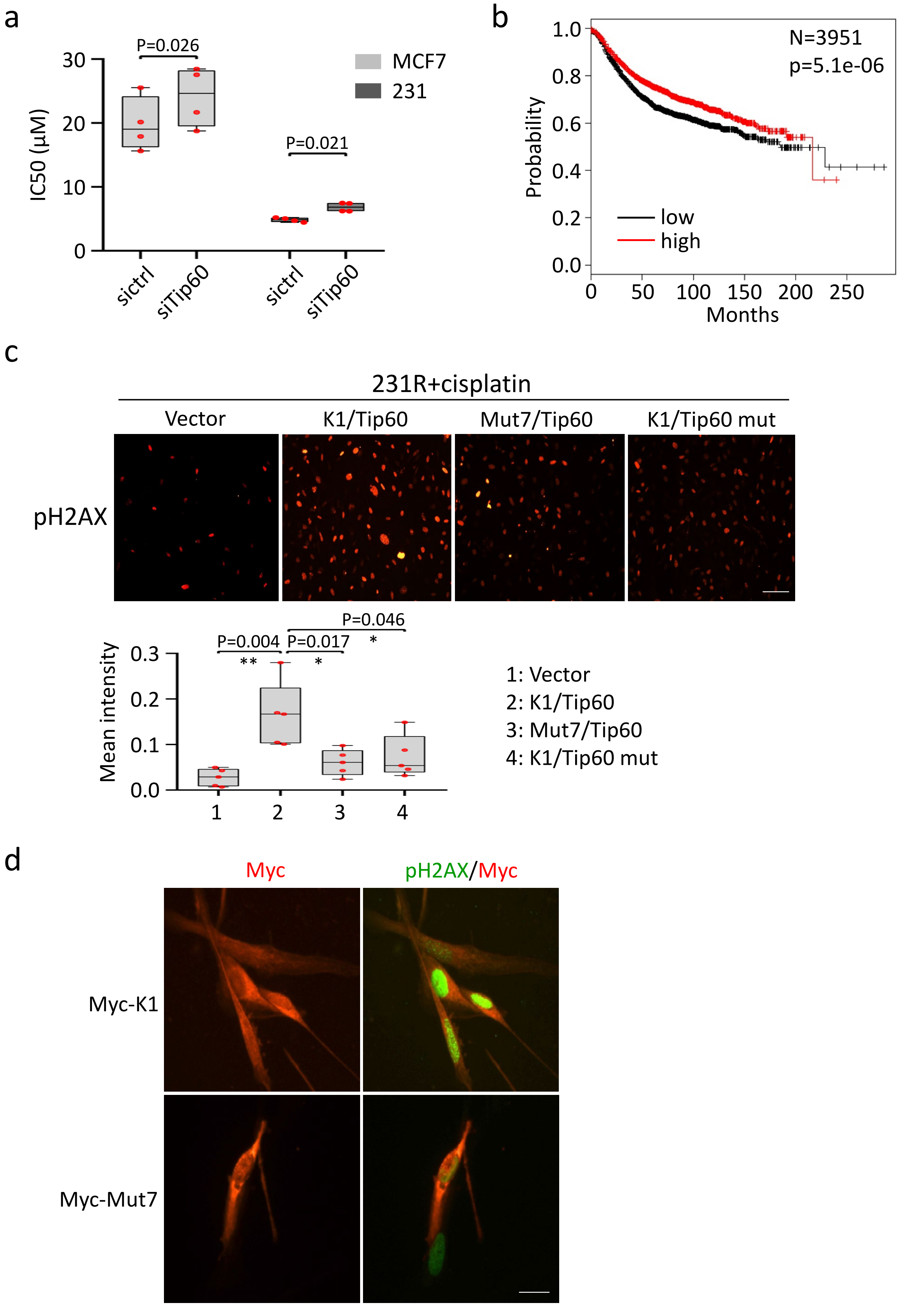

Supplementary Figure 16

Supplementary Figure 16. SRPK1 acetylation affected cellular response to cisplatin. (**a**) After Tip60 was knocked down in MCF7 and 231 cells, the IC50 of cisplatin was determined by MTS viability assays. Bars: mean ± SD; n=3; *p<0.05 by Student's t-test. (**b**) Kaplan-Meier analyses of the survival probability of 3,951 breast cancer patients grouped by the high or low expression of Tip60. (**c**) 231R cells were transfected with SRPK1 and Tip60 constructs as indicated, and treated with cisplatin. The cells were then immunostained for pH2AX. Scale bar: 100 μm. The mean fluorescence intensity was quantified from five random fields using Image J. Boxplot: n=5; *p<0.05, **p<0.01 by Student's t-test. (**d**) 231R cells were transfected with Myc-tagged SRPK1 or acetylation-deficient Mut7 and treated with cisplatin. The cells were double-stained for Myc and pH2AX. Scale bar: 20 μm. The images are representative of three experiments with similar results.

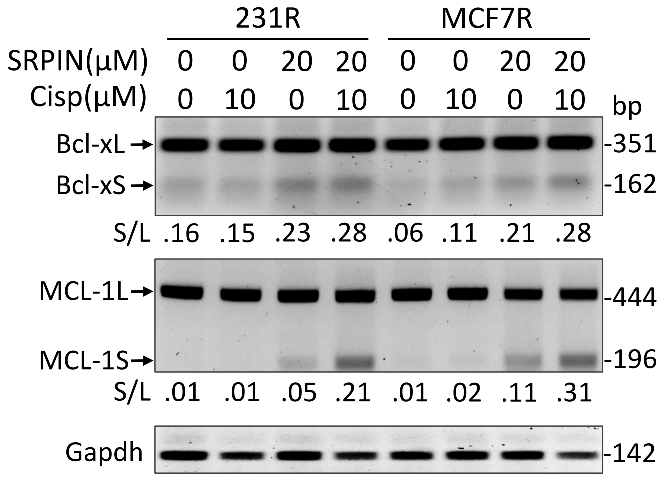

Supplementary Figure 17. Inhibition of SRPK1 activity could favor pro-apoptotic splicing. 231R and MCF7R were co-treated with cisplatin and SRPIN340 as indicated. The alternative splicing of *BCL2L1* and *MCL-1* was assessed by RT-PCR. The decimals below the gel strips denote the relative abundance of short (S) versus long (L) variants. The RT-PCR gel images are representative of three experiments with similar results.

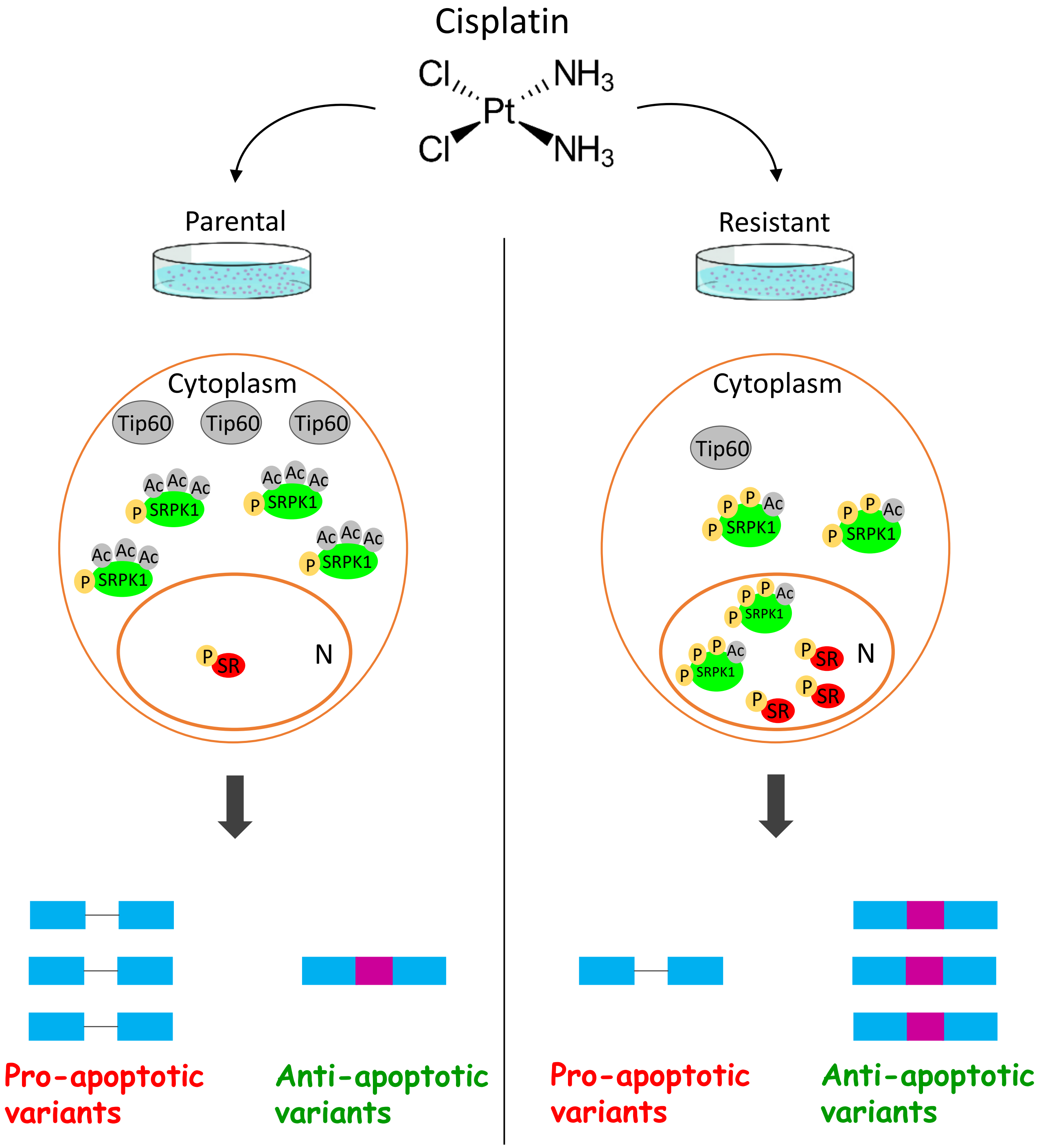

Supplementary Figure 18. The putative model. N: nucleus. P: phosphorylation. Ac: acetylation.

Supplementary Table 1: Primers used in RT-PCR and cloning of shRNA targeting SRPK1

Name	Sequence
MCL1-F	atctctcggtaccttcgggagc
MCL1-R	cctgatgccaccttctaggtcc
BCL2L1-F	catggcagcagtaaagcaag
BCL2L1-R	gcattgttcccatagagttcc
BARD1-F	gaggagcctttcatccgaaggc
BARD1-R	gctctcacaaaccgtgcaaa
SRPK1-F	gtgtgccagtcttcctcaactg
SRPK1-R	ggtcagcaatcttcaccttgag
KAT5-F	gtttcaccagcaactccagtgc
KAT5-R	acggtattccatcagagctctcc
VEGF-F	tttgtttgtacaagatccgcagacg
VEGF-R	tcgttctgtatcagtctttcctgg
BAX-F	cggggagcagcccaga
BAX-R	aaagtaggagaggaggccgt
GAPDH-F	aacatcatccctgcctctactgg
GAPDH-R	gtttttctagacggcaggtcagg
shSRPK1 sense	ccggcccattaggacatcctttaaactgcagtttaaaggatgtcctaatgggttttt
shSRPK1 antisense	aattaaaaacccattaggacatcctttaaactgcagtttaaaggatgtcctaatggg

F: forward primer; R: reverse primer

Uncropped blot and gel images

Fig 1c

SRPK1: MCF7/R

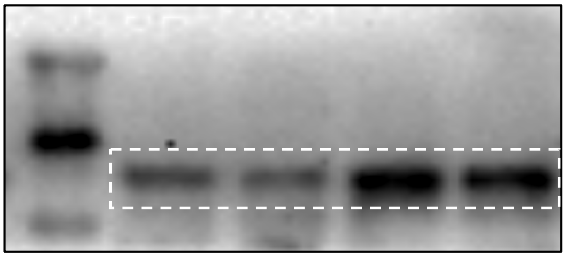

SRPK1: 231/R

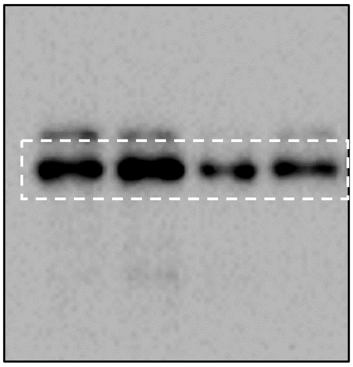

Actin: MCF7/R

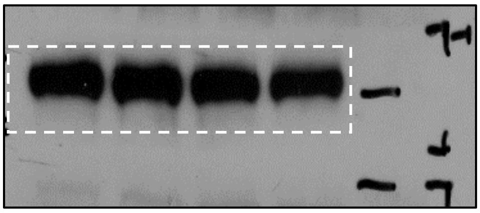

Actin: 231/R

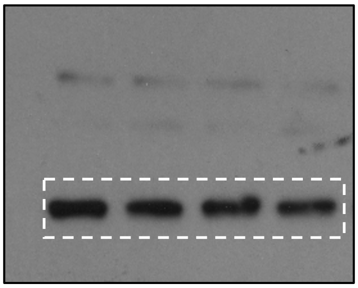

Fig 1d

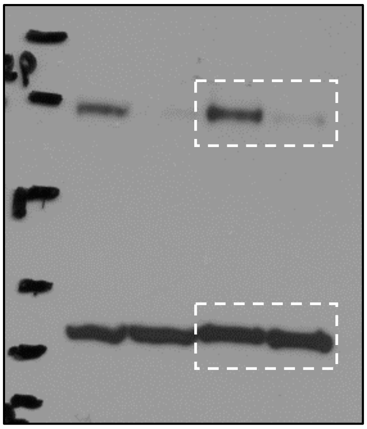

siK1: SRPK1

siK1: Actin

Myc-K1: Myc

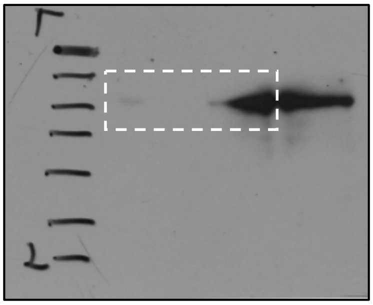

Myc-K1: SRPK1

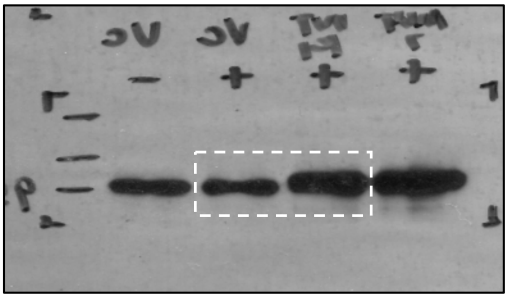

Myc-K1: Actin

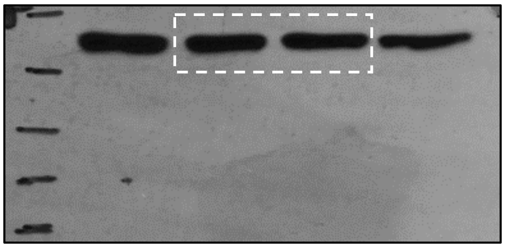

Fig 2a

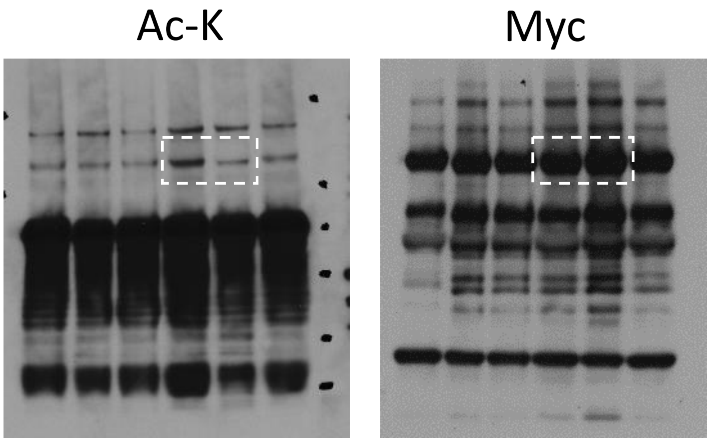

Fig 2b

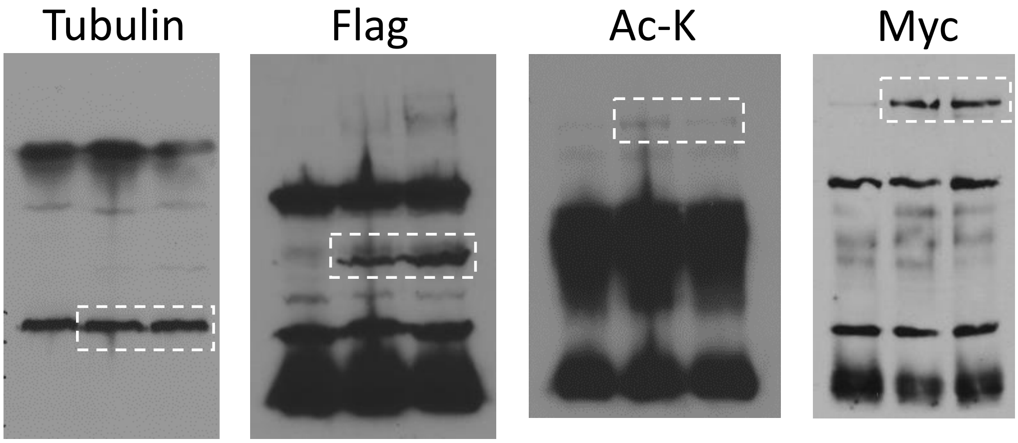

Fig 2c

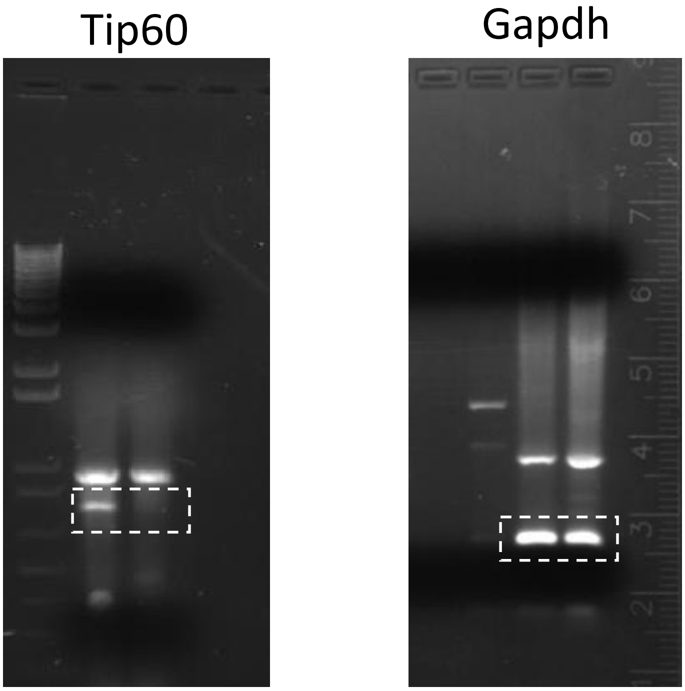

Fig 2e

SRPK1

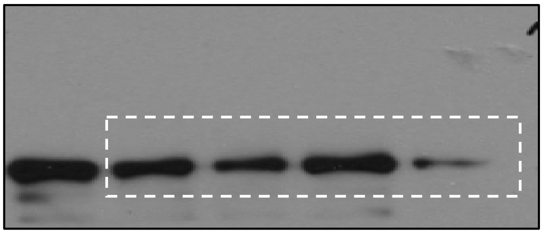

Actin

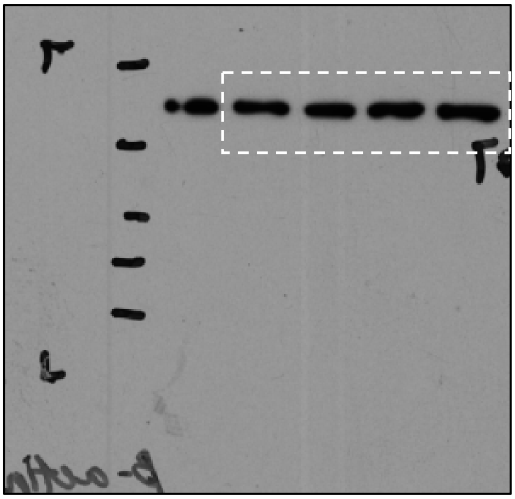

Tip60

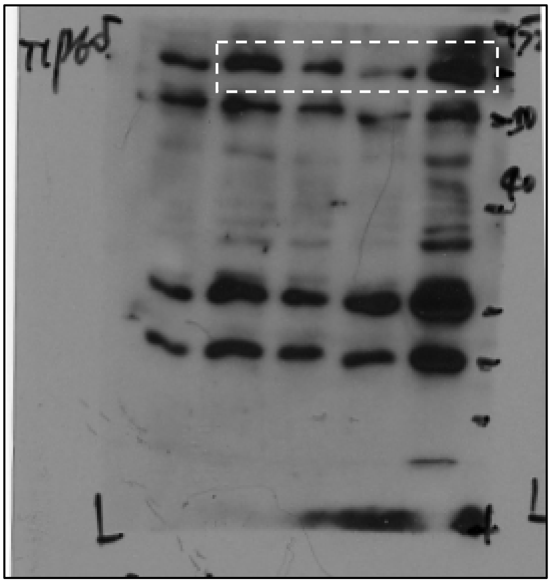

Fig 2f

Ac-K left

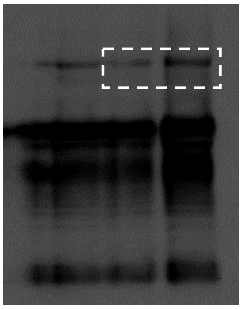

Ac-K right

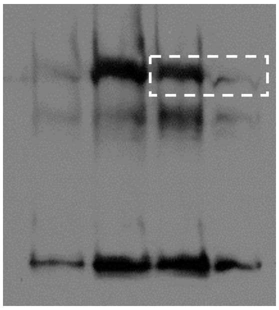

SRPK1 left

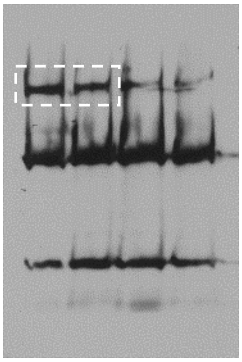

SRPK1 right

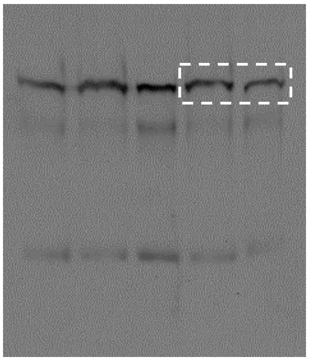

Fig 2g

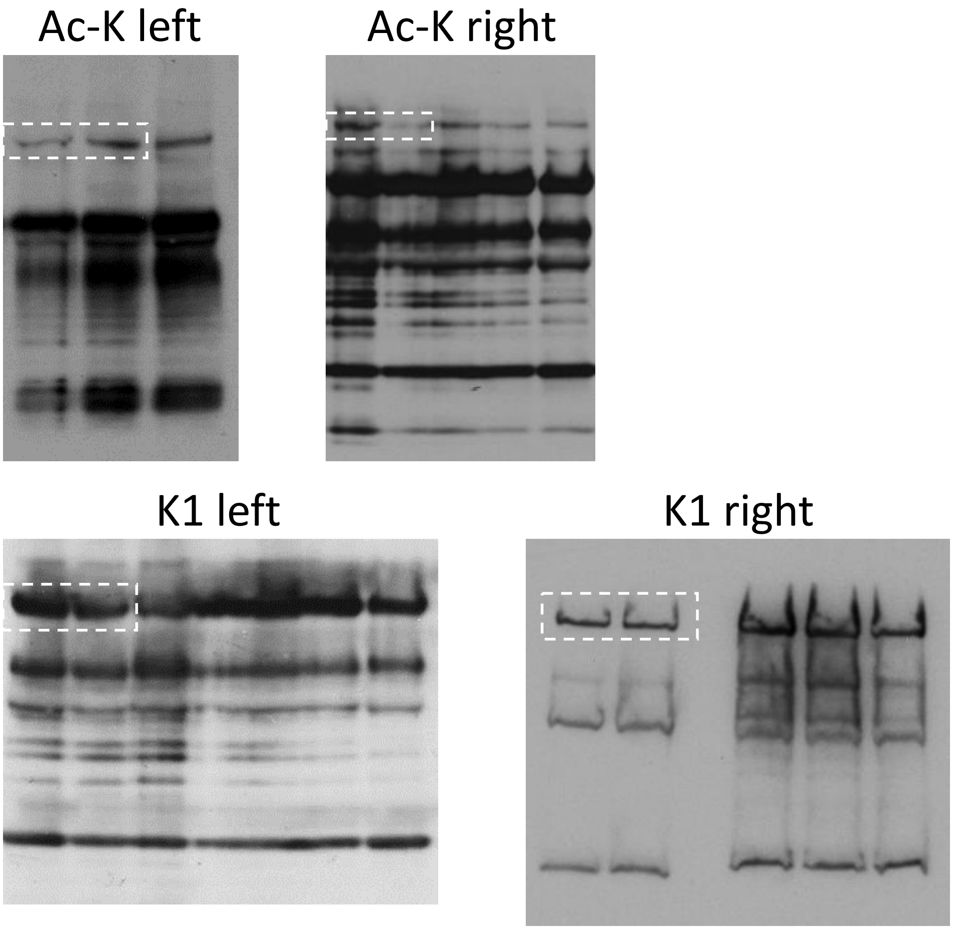
Ac-K left
Ac-K right
K1 left
K1 right

Fig 2h

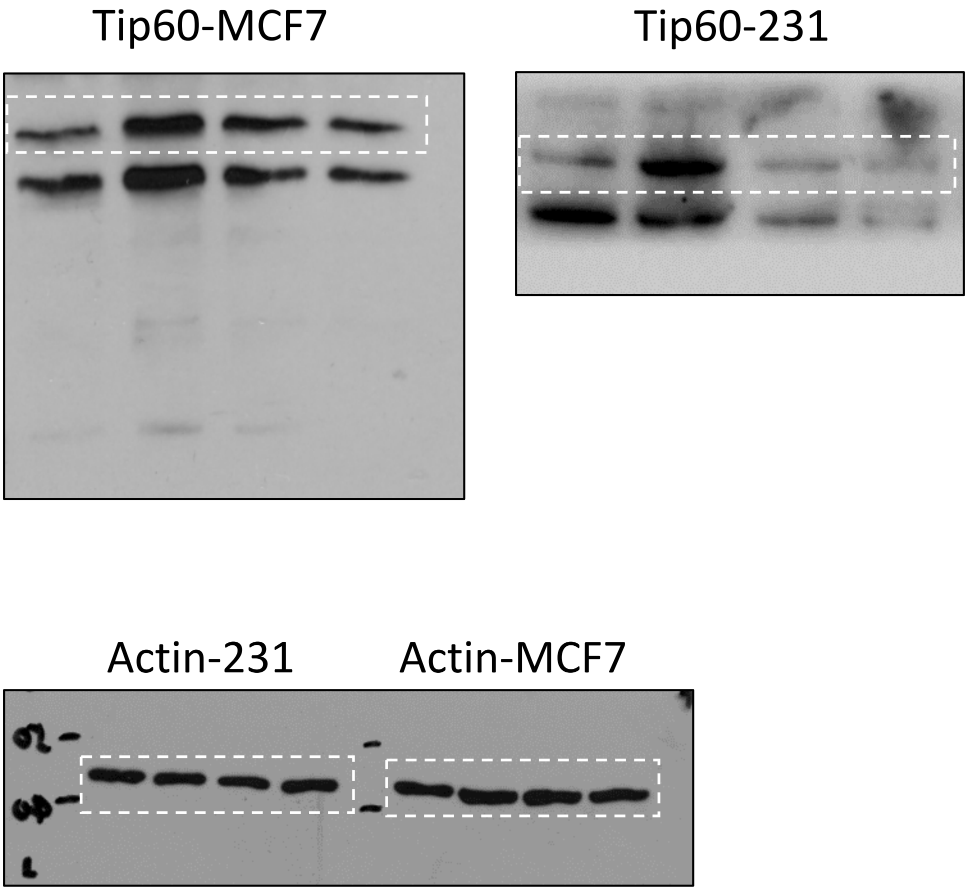
Tip60-MCF7
Tip60-231
Actin-231
Actin-MCF7

Fig 2i

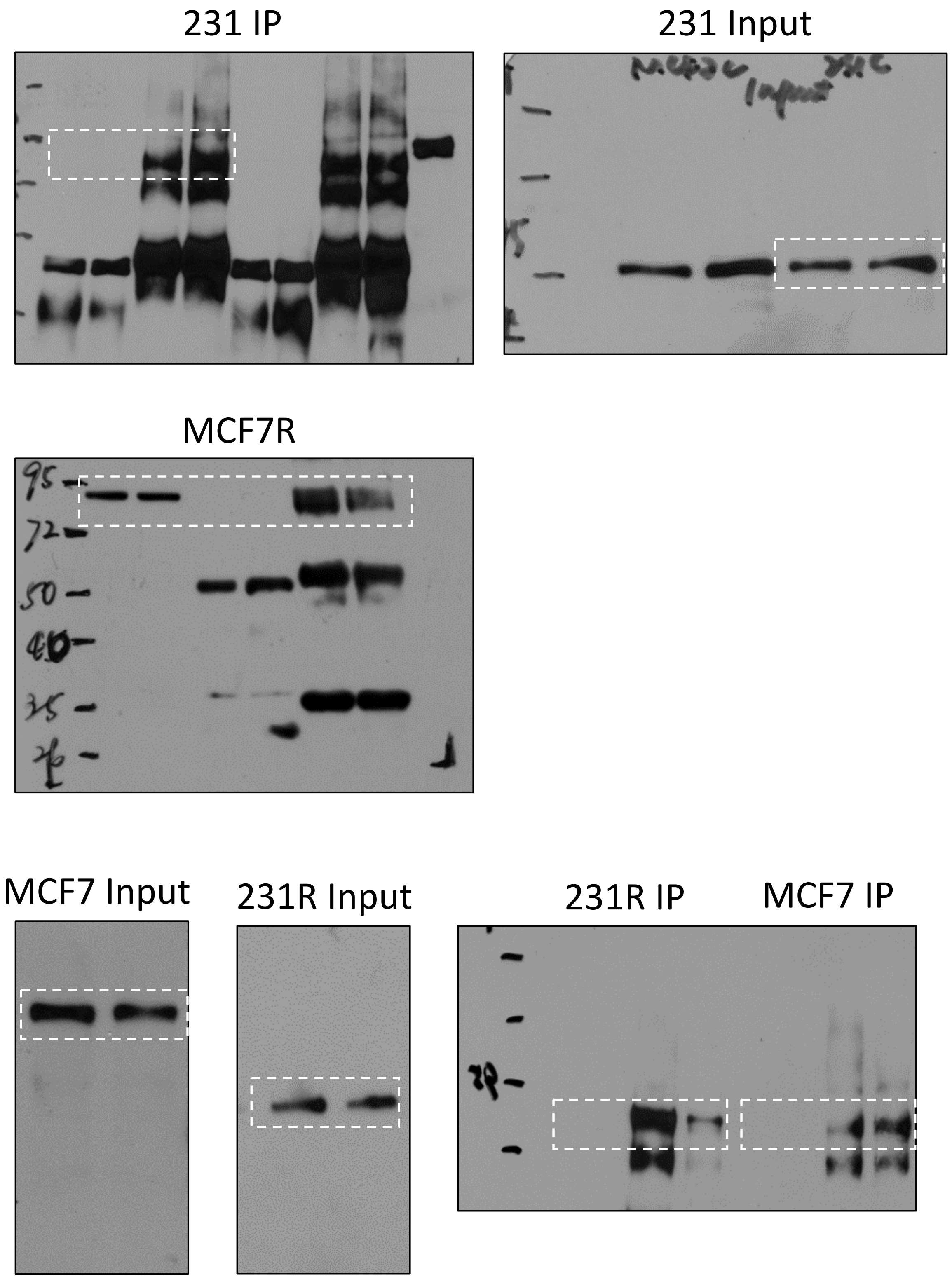

Fig 3a

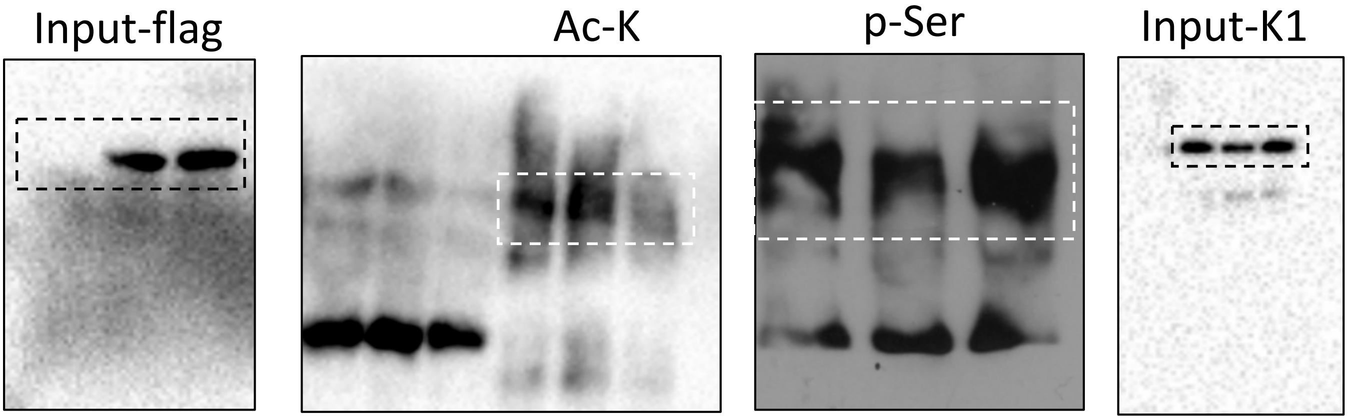

Fig 3b

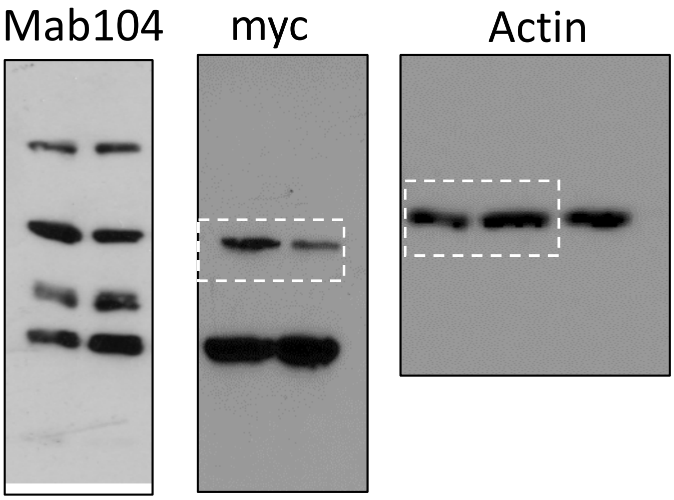

Fig 3c

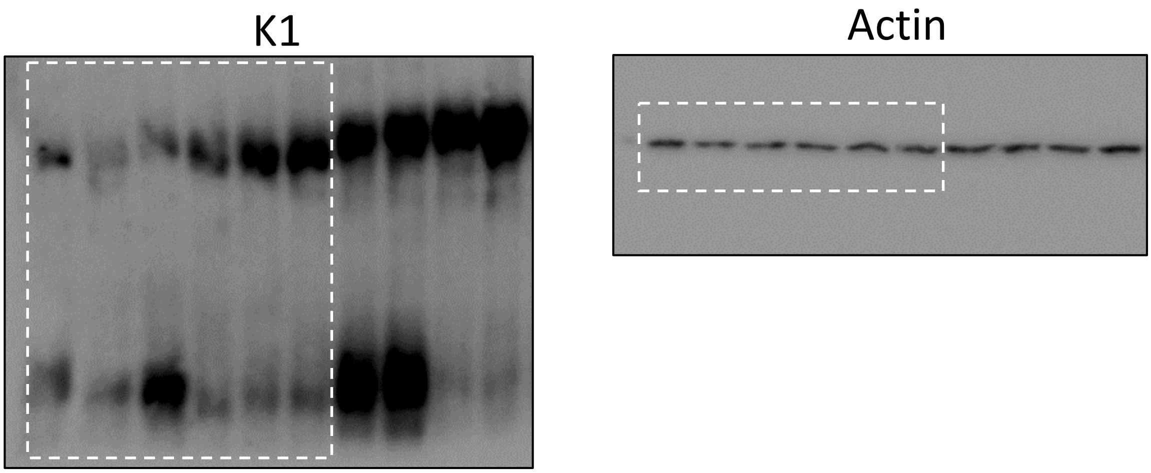

Fig 3d

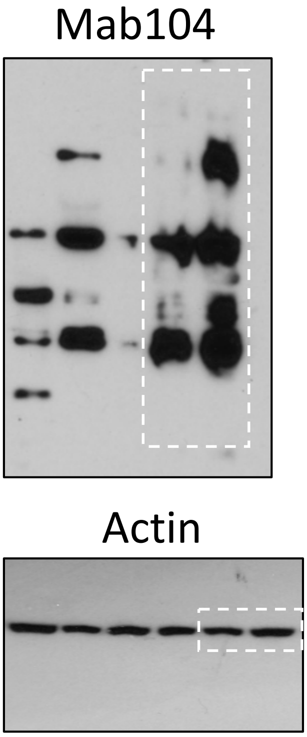

Fig 3e

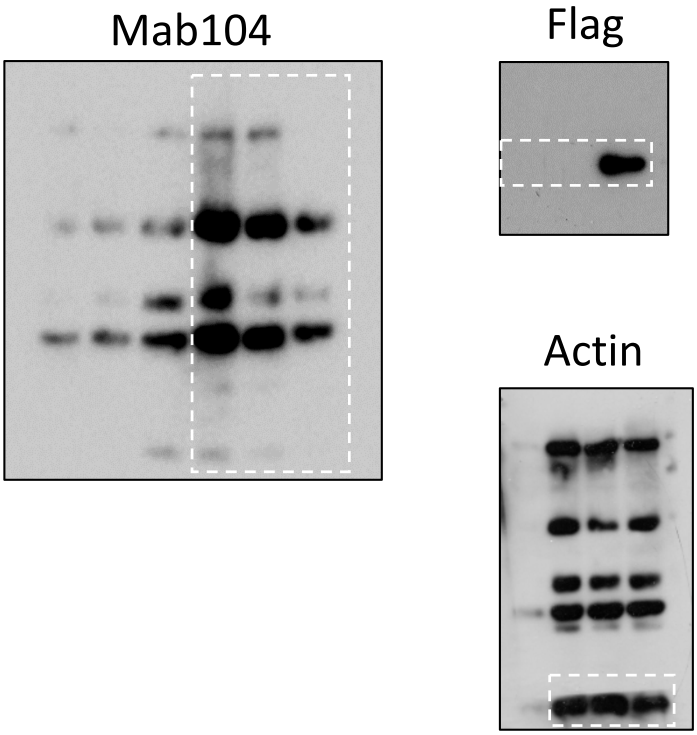

Fig 3f

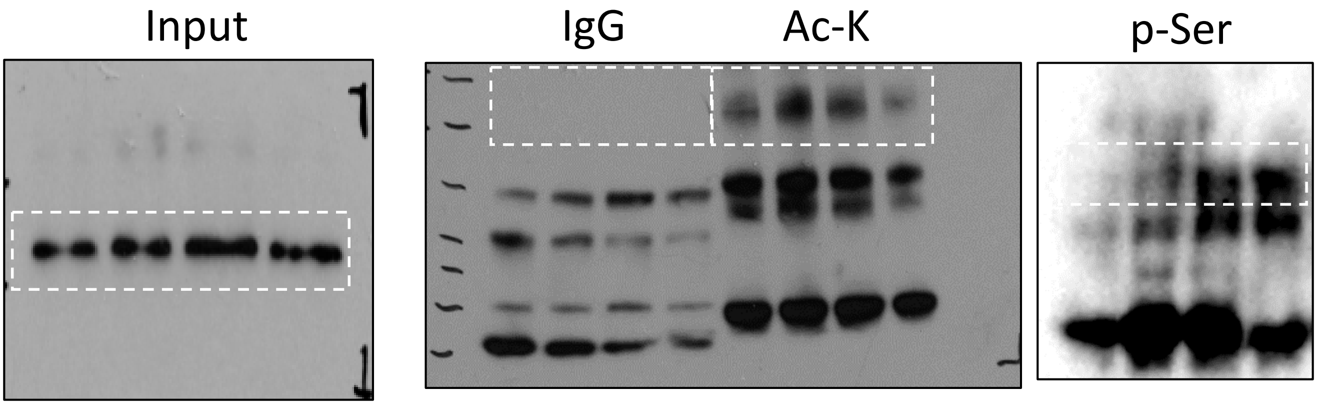

Fig 3g

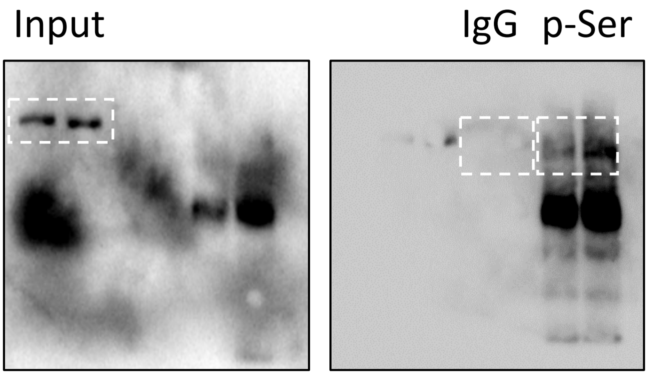

Fig 3h

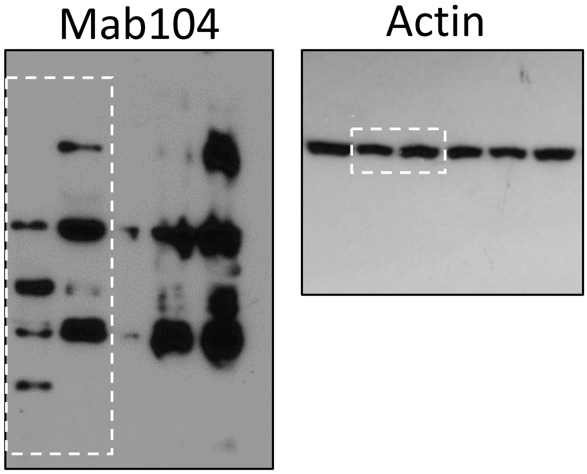

Fig 3i

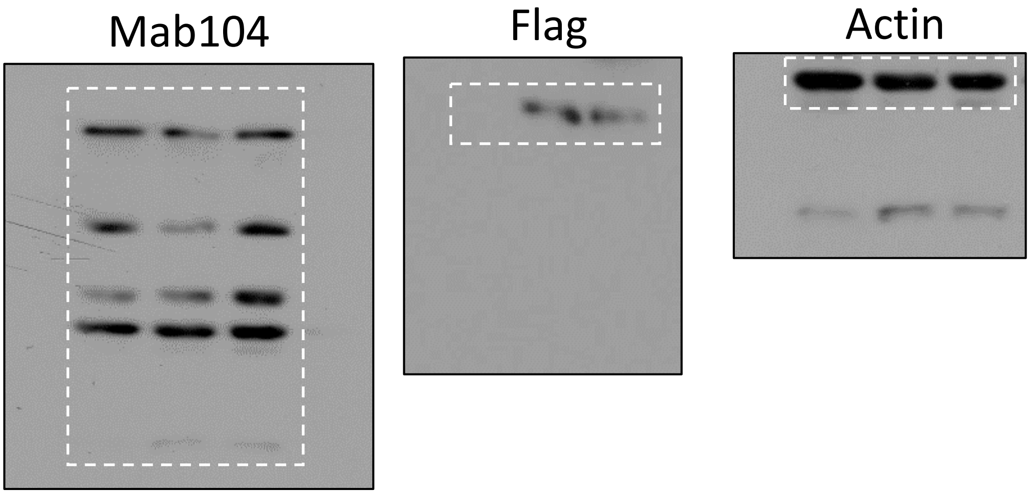

Fig 3j

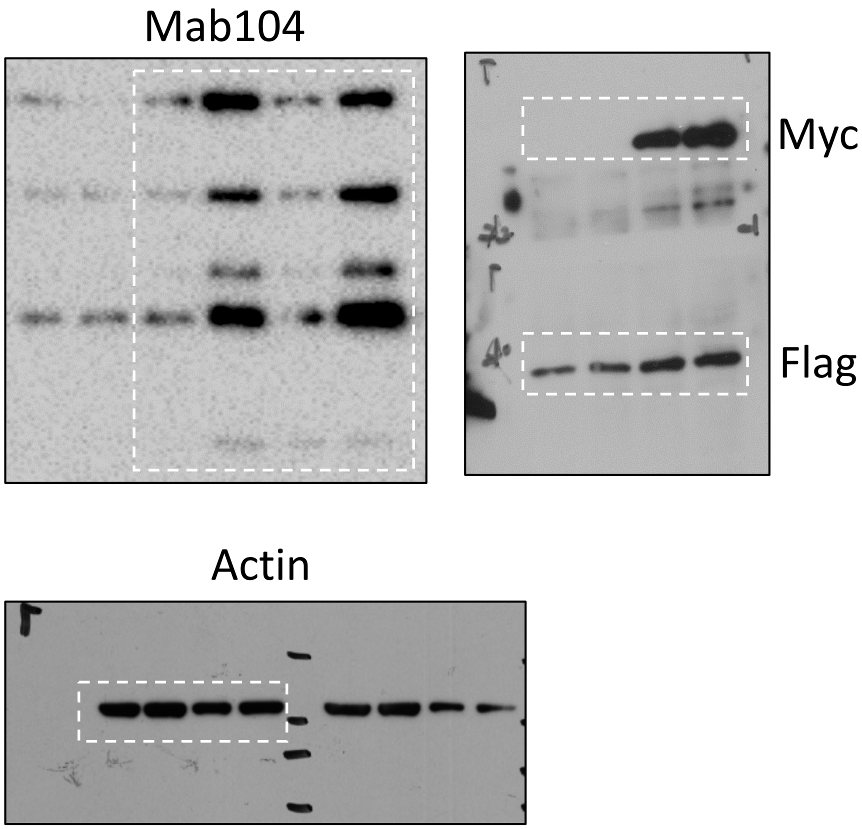

Fig 4b

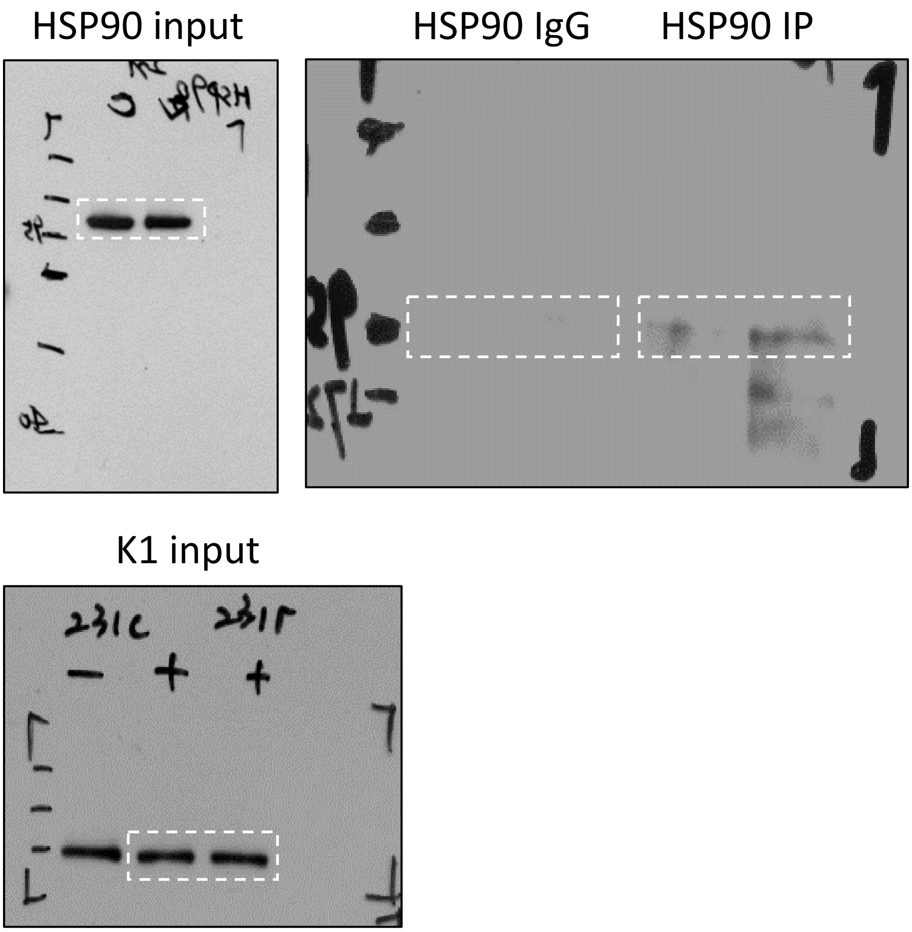

Fig 4c

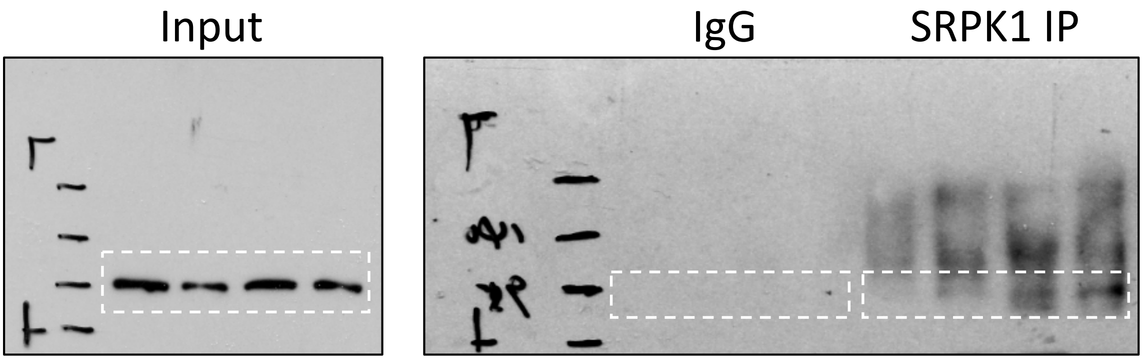

Fig 4e

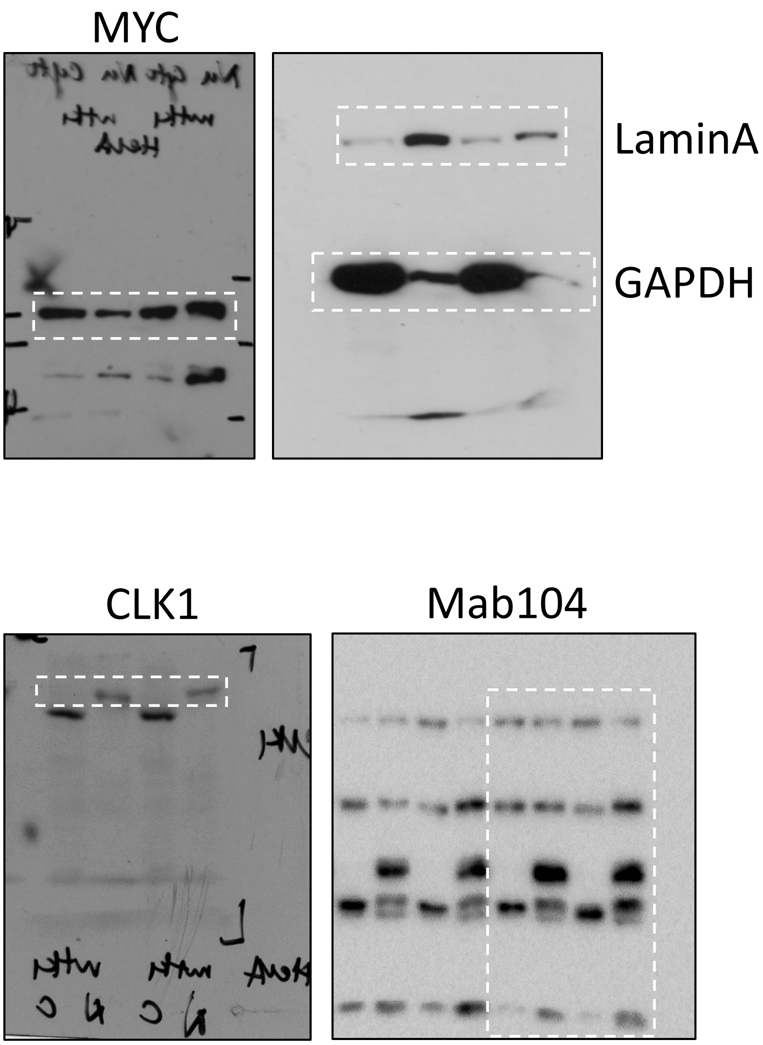

Fig 5a

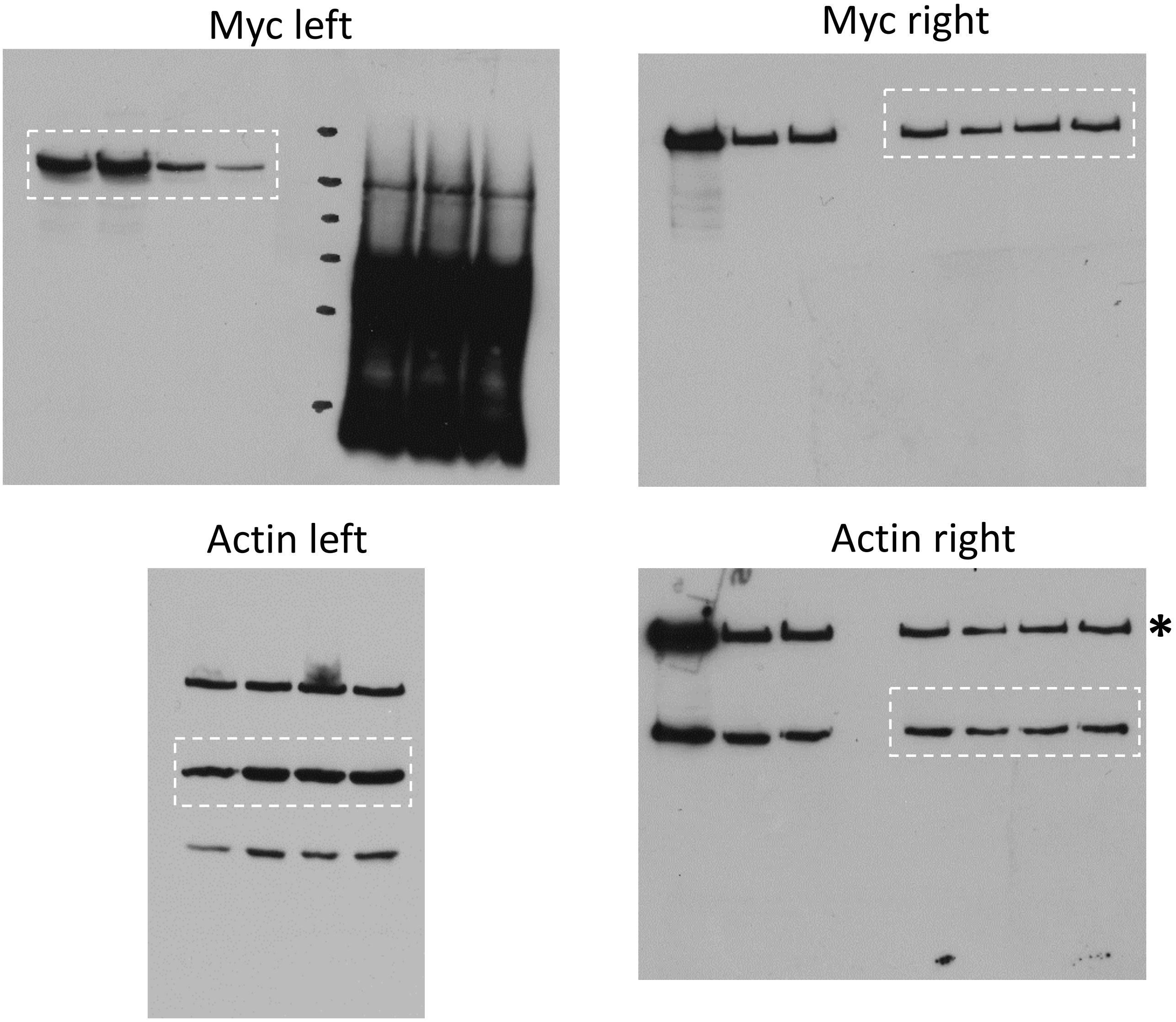

* Incomplete stripping of anti-Myc signals carried over from the blot above

Fig 5b

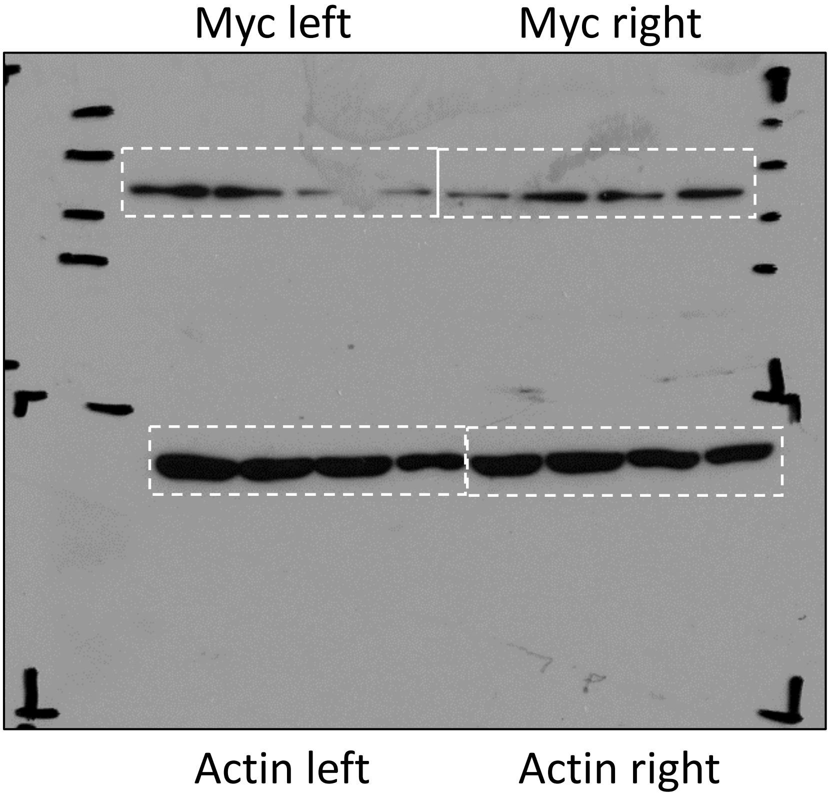
Myc left
Myc right
Actin left
Actin right

Fig 5d

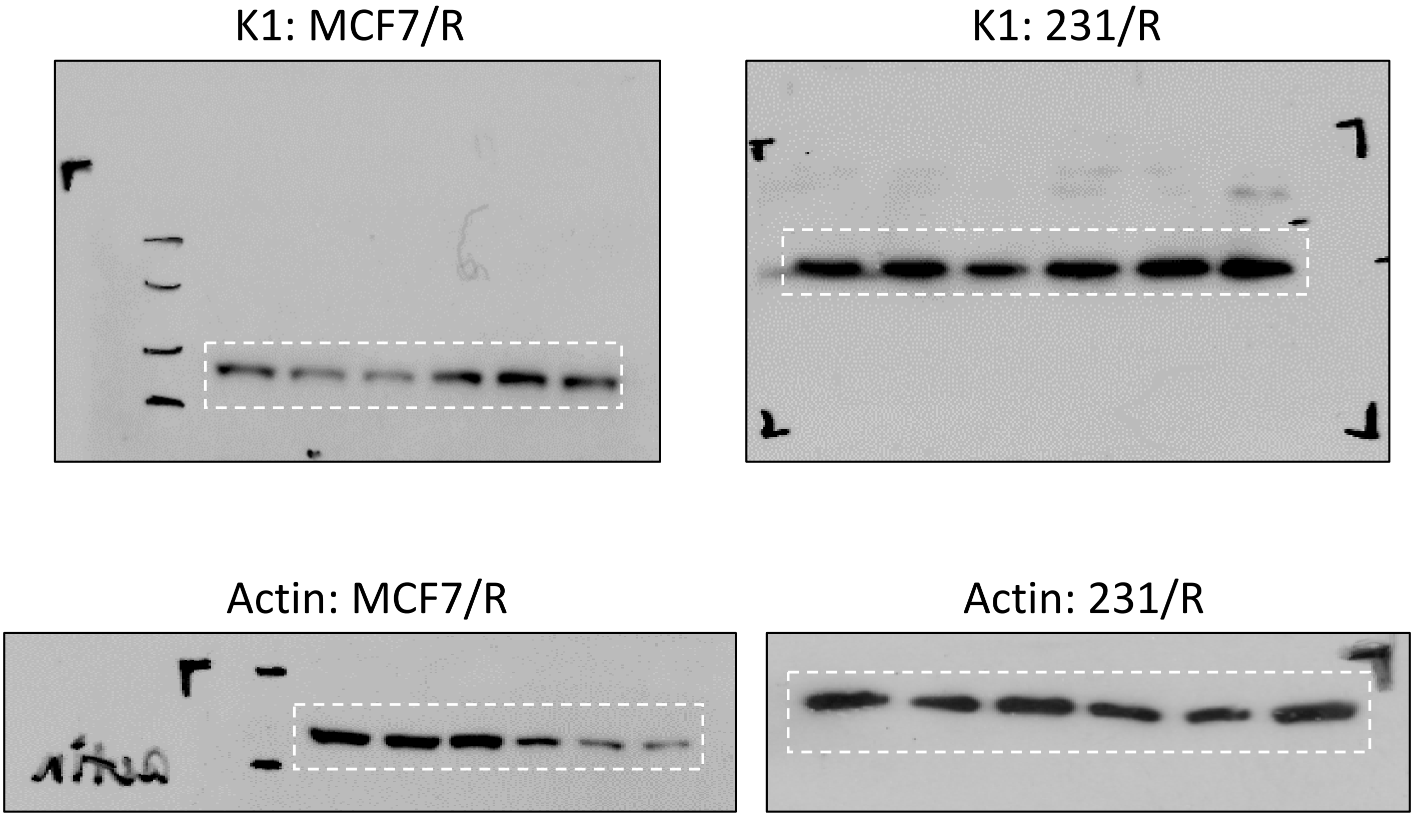
K1: MCF7/R
K1: 231/R
Actin: MCF7/R
Actin: 231/R

Fig 6a

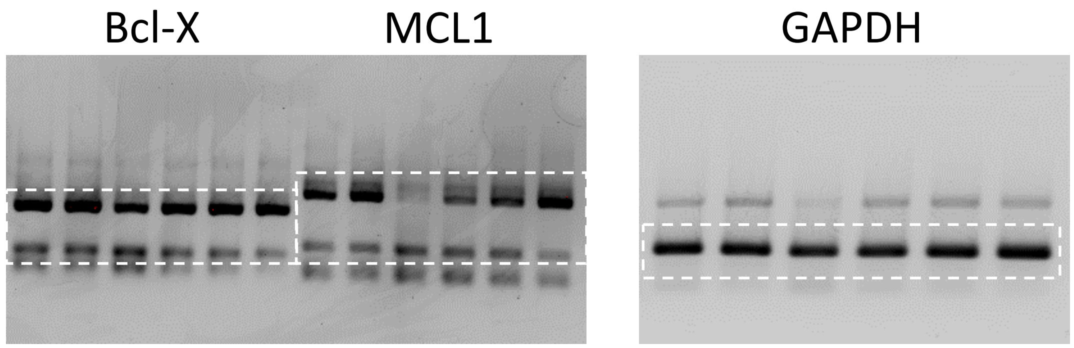

Fig 6b

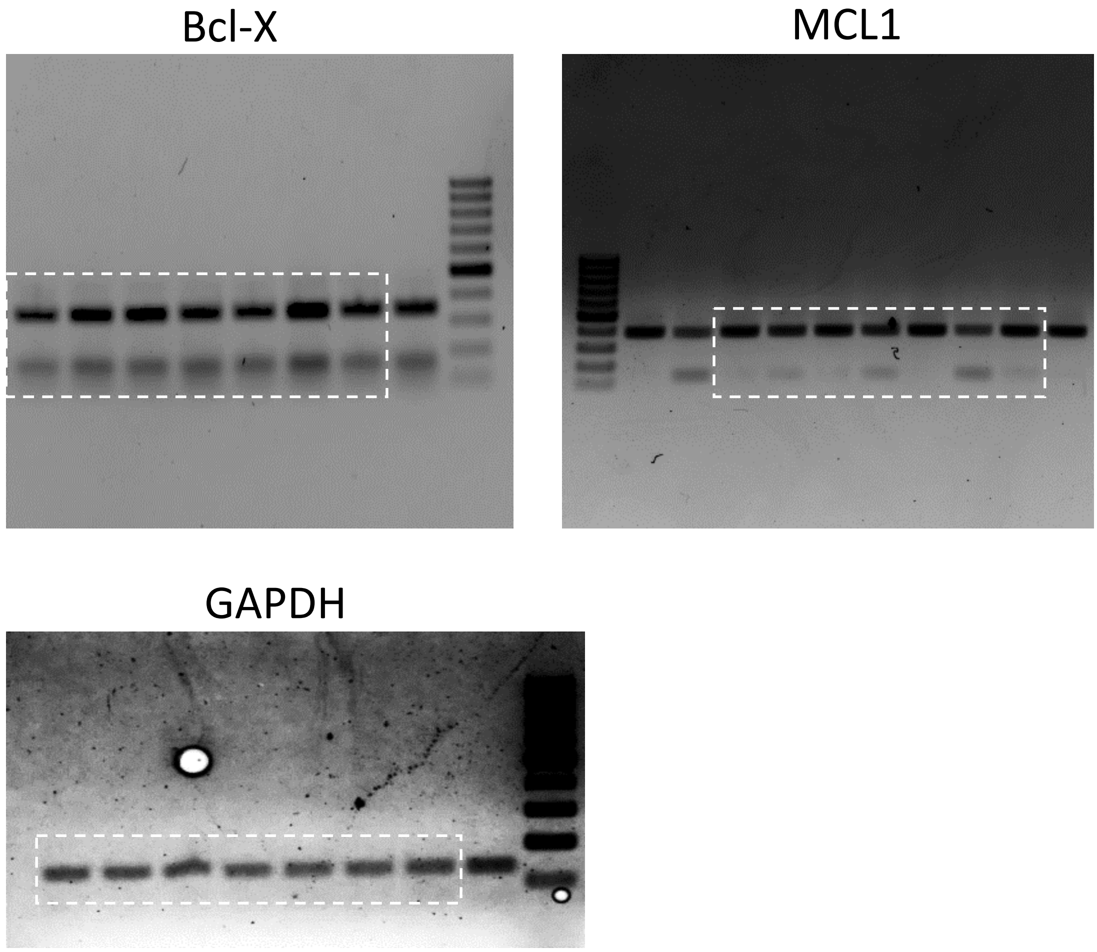

Fig 7c

PARP1

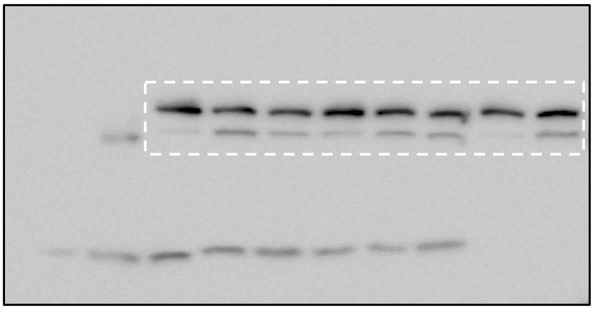

K1

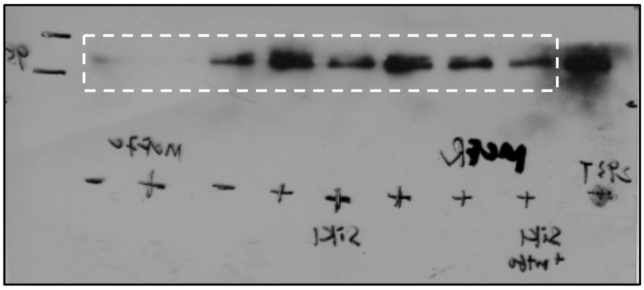

FLAG

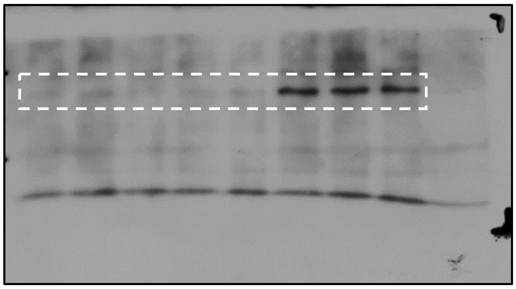

Actin

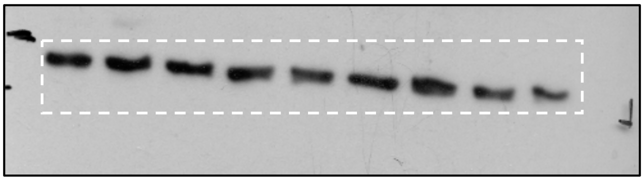

Fig 7d

PARP1

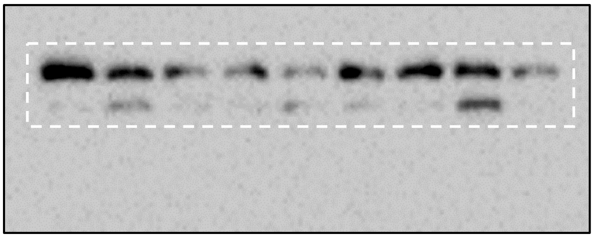

MYC

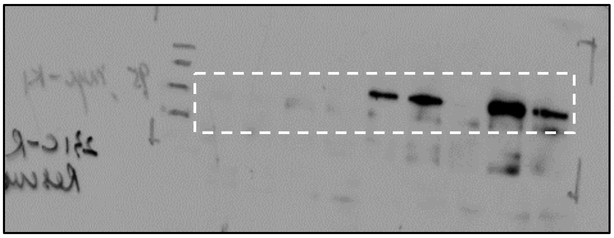

FLAG

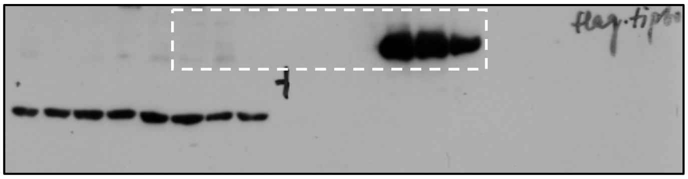

Actin

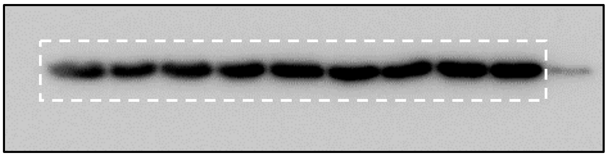

Fig 8a

Mab104

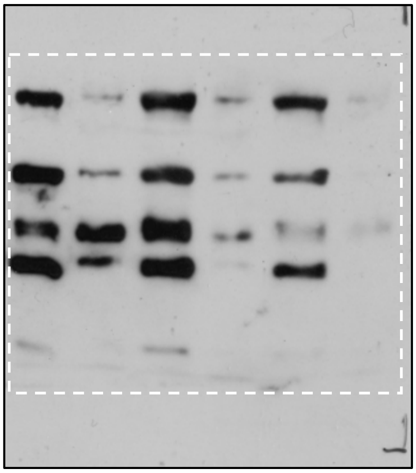

GAPDH

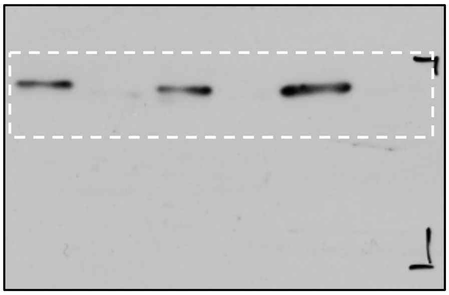

LaminA

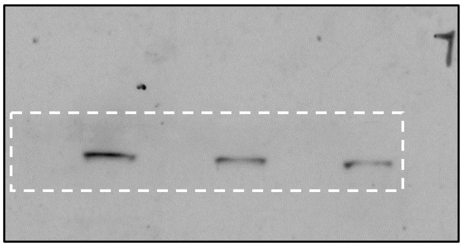

Fig 8c

PARP1

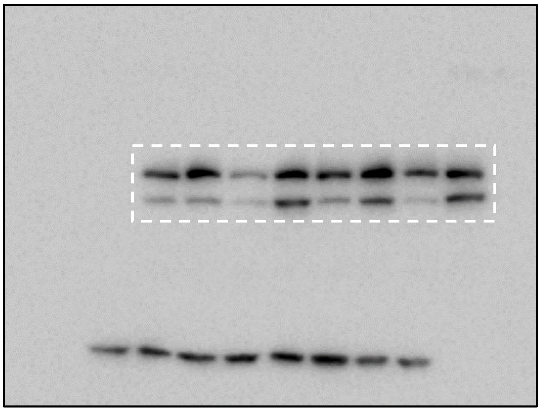

Actin

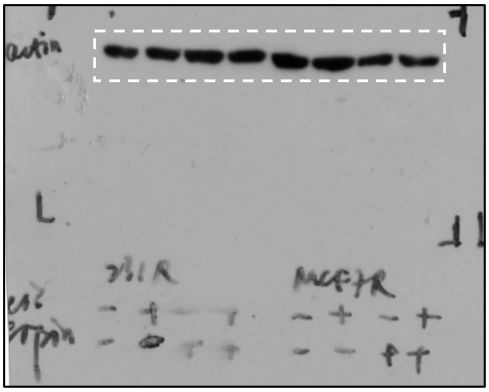

Fig 8d

Bcl-X

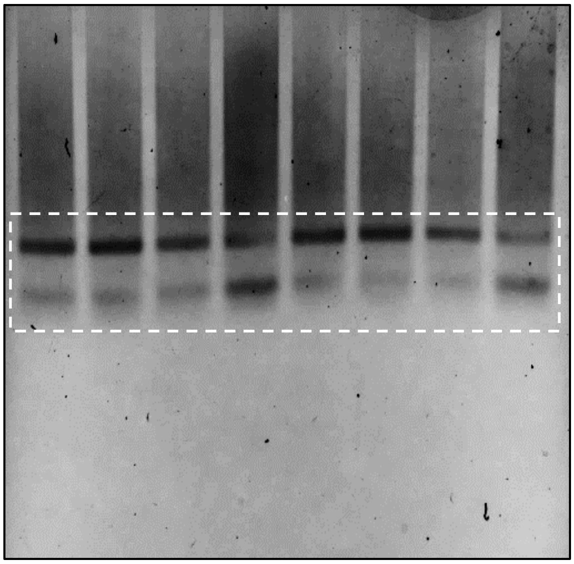

MCL-1

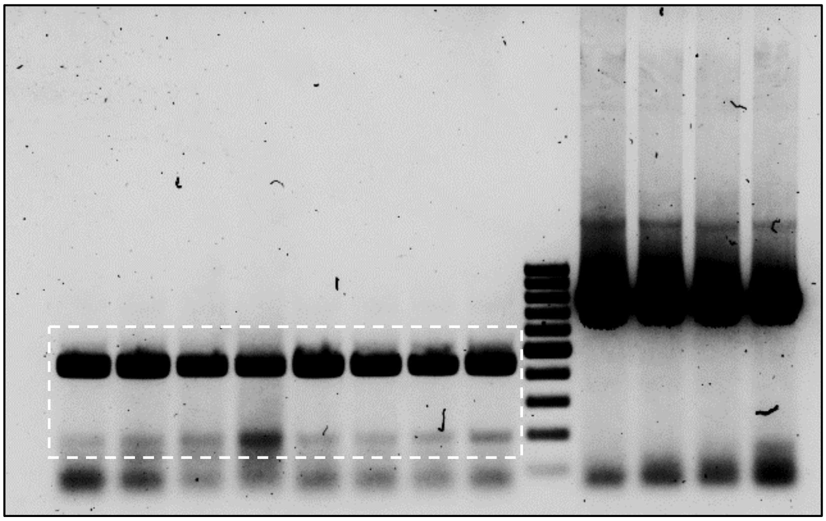

GAPDH

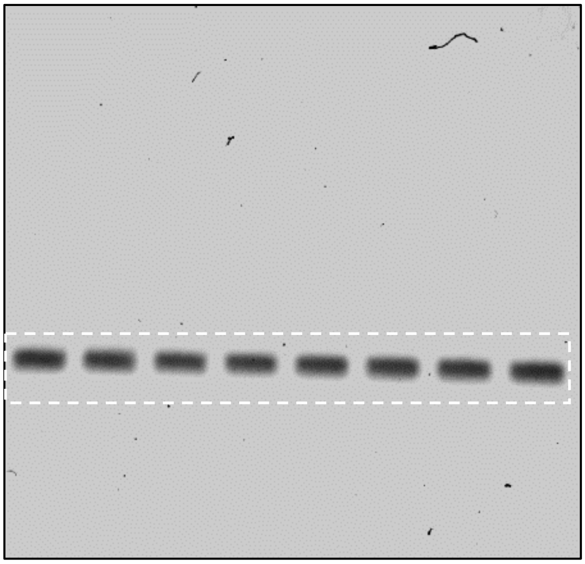

Sup Fig 2

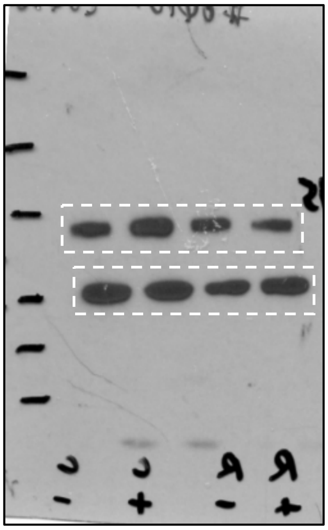

231: SRPK1

231: Actin

Sup Fig 3a

SRPK1: MCF7/R

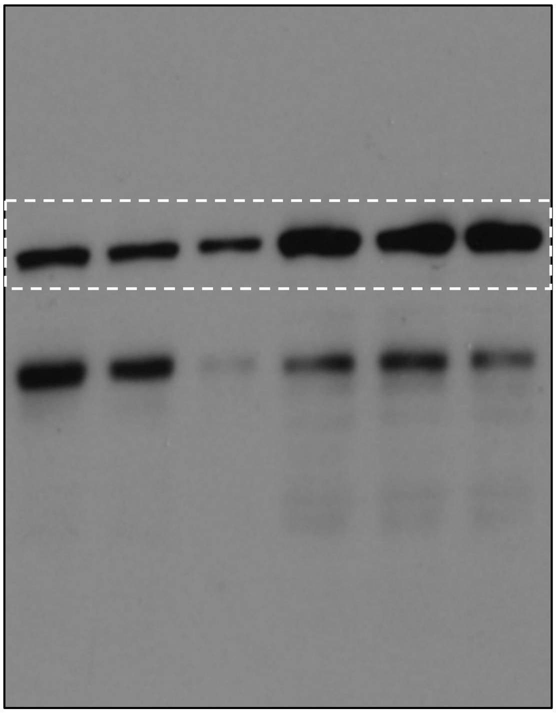

SRPK1: 231/R

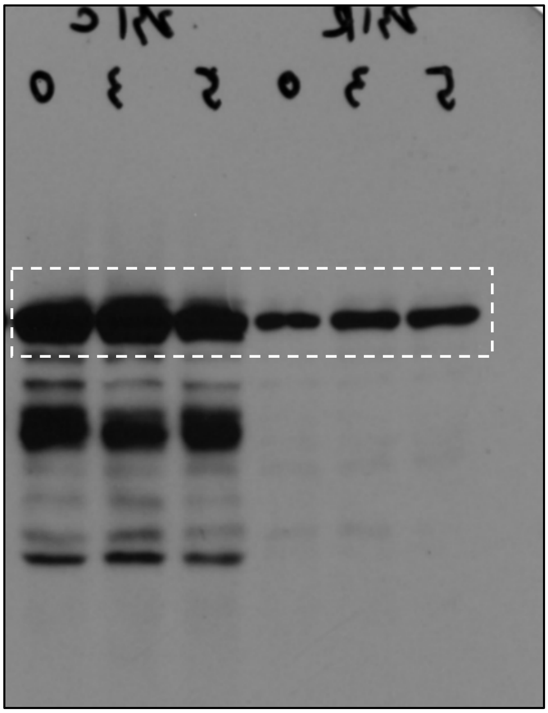

Actin: MCF7/R Actin: 231/R

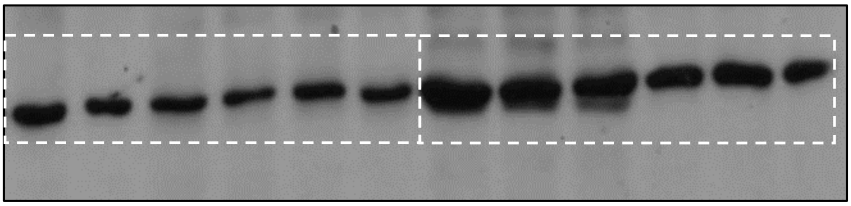

Sup Fig 6c

TIP60: carboplatin TIP60: oxaliplatin

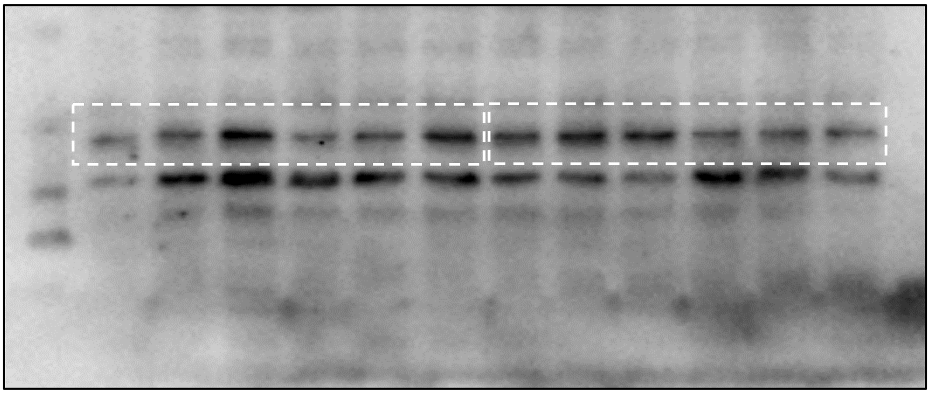

ACTIN: carboplatin ACTIN: oxaliplatin

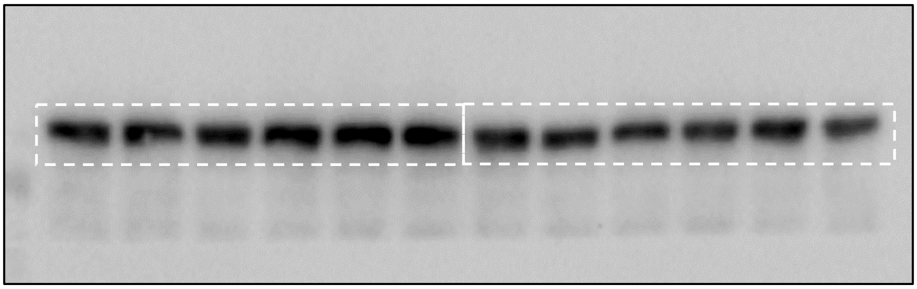

Sup Fig 7

AKT- S473

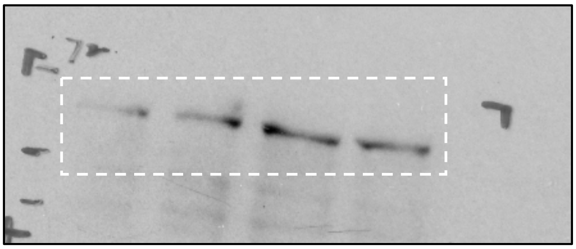

mTOR

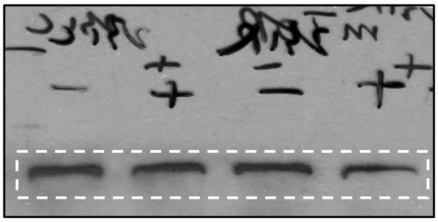

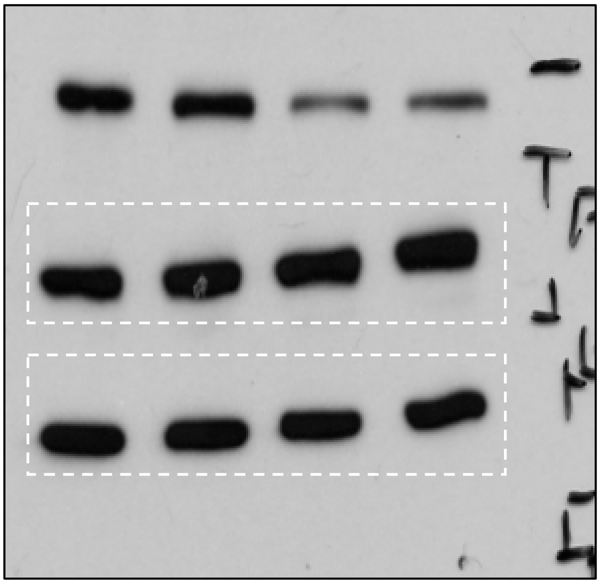

AKT

ACTIN

Sup Fig 8b

SRPK1

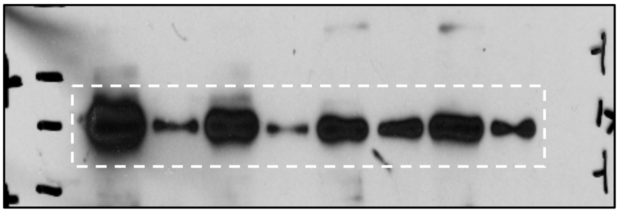

Lamin A

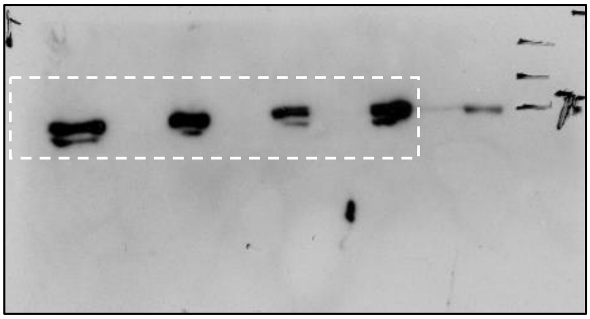

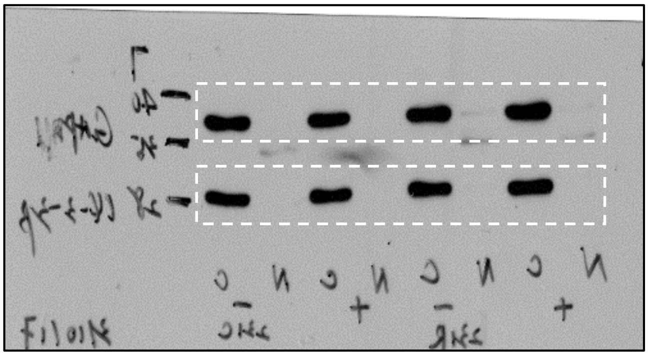

GAPDH

14-3-3

Sup Fig 9c

MYC

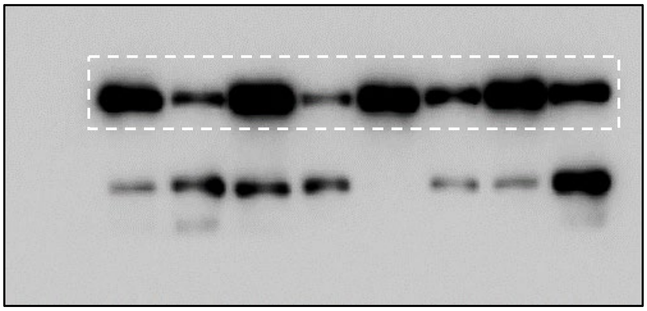

FLAG

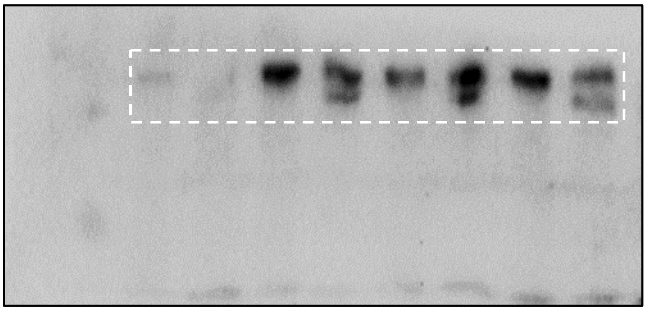

GAPDH

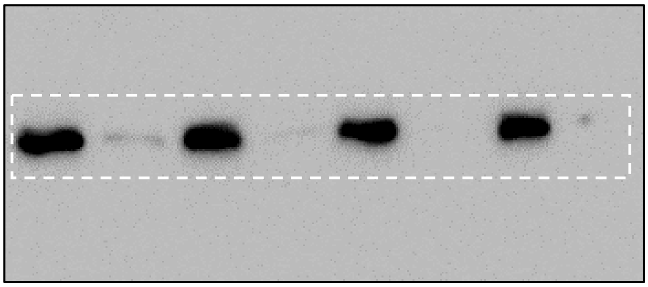

LaminA

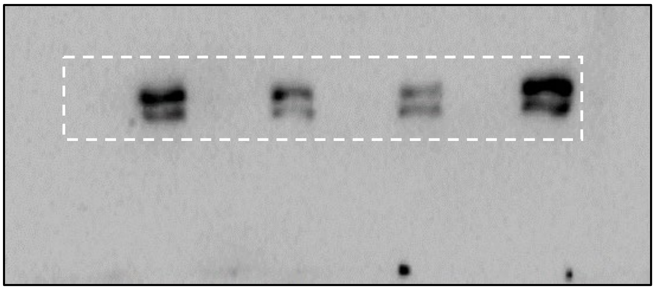

Sup Fig 11b

BARD1

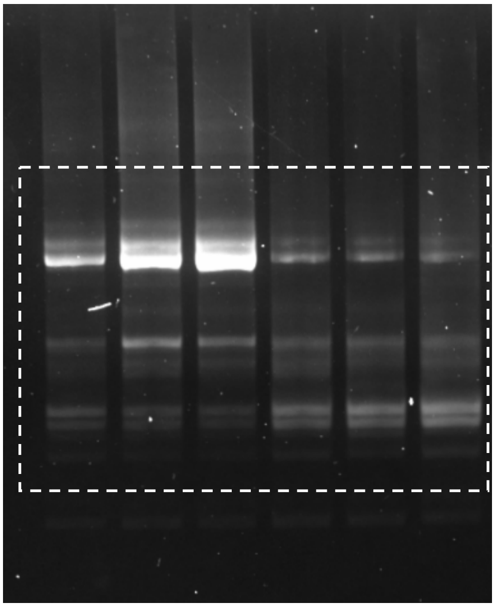

GAPDH

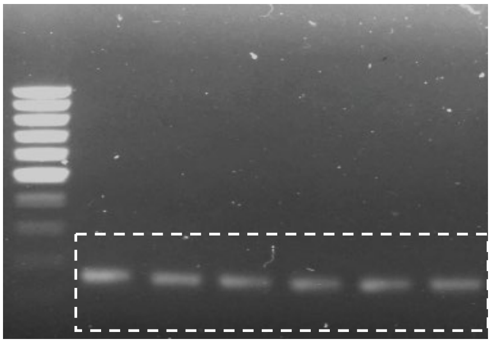

Sup Fig 11c

BARD1

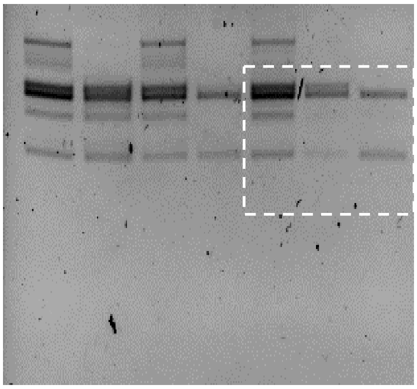

GAPDH

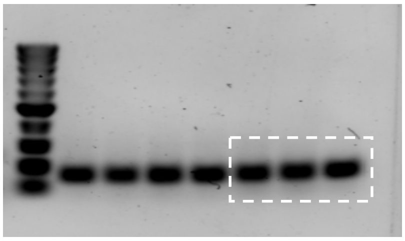

Sup Fig 11d

BARD1

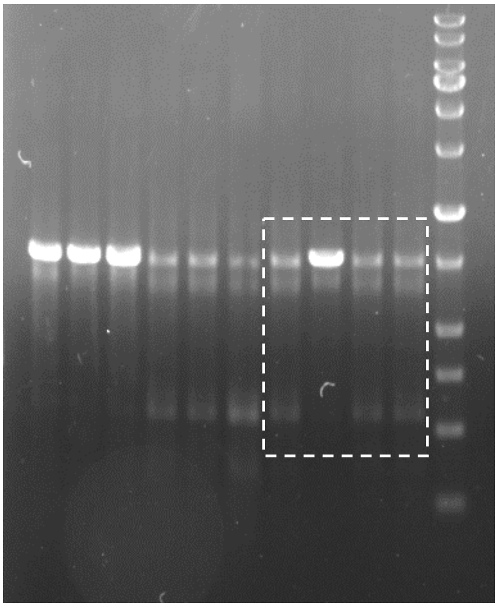

GAPDH

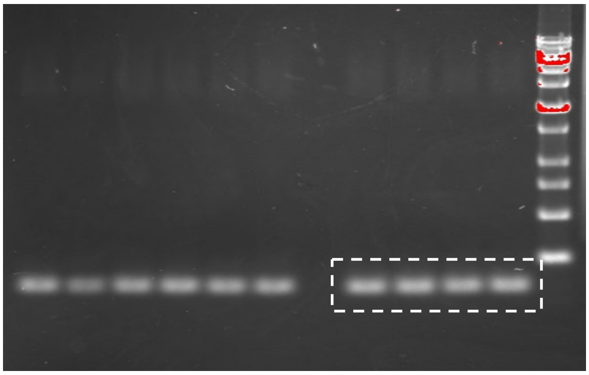

Sup Fig 12c

Bcl-X

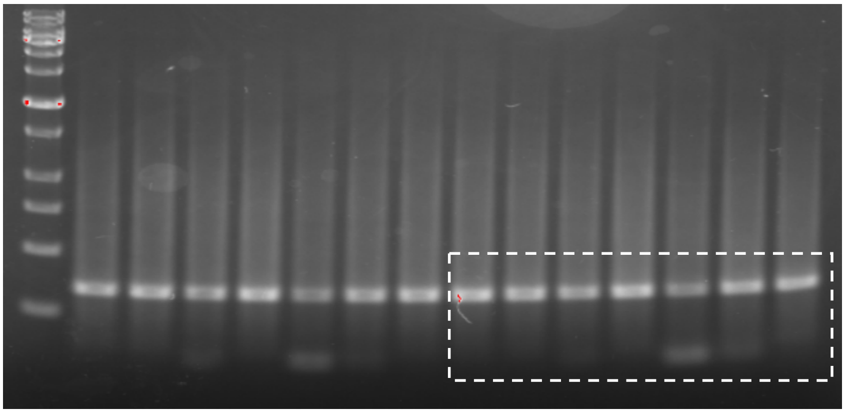

MCL-1

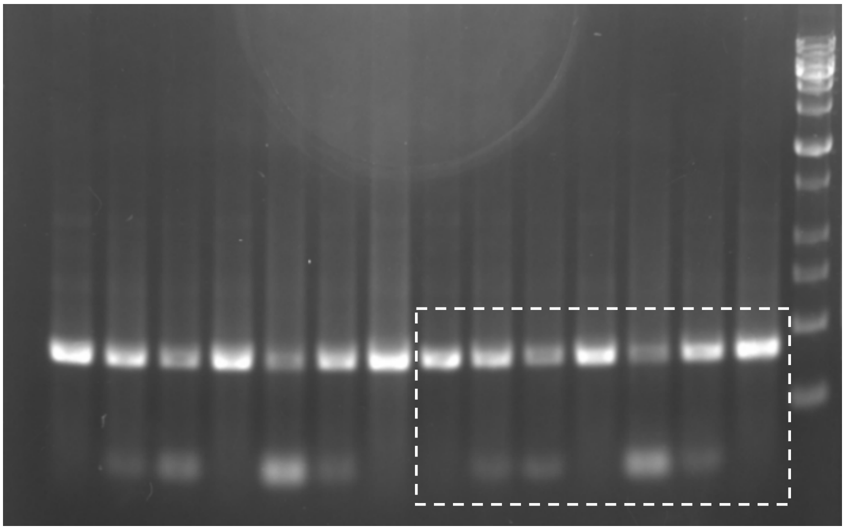

GAPDH

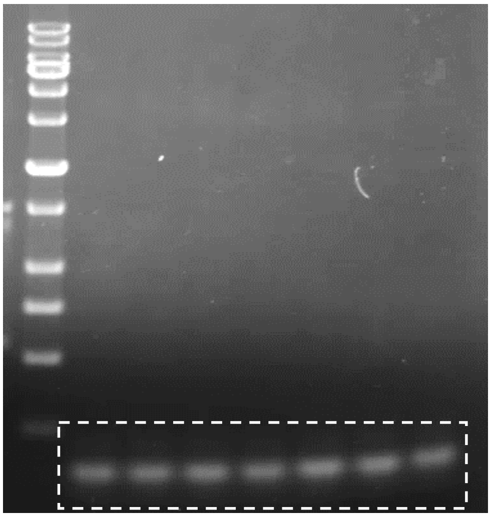

Sup Fig 13b

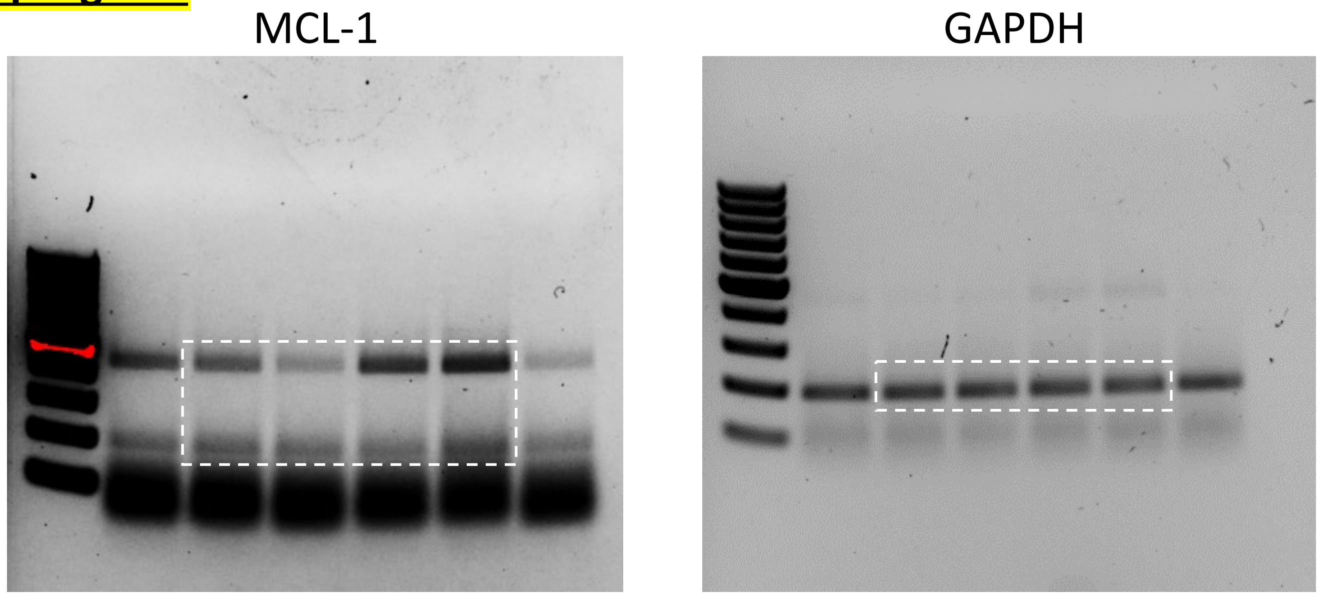

Sup Fig 13c

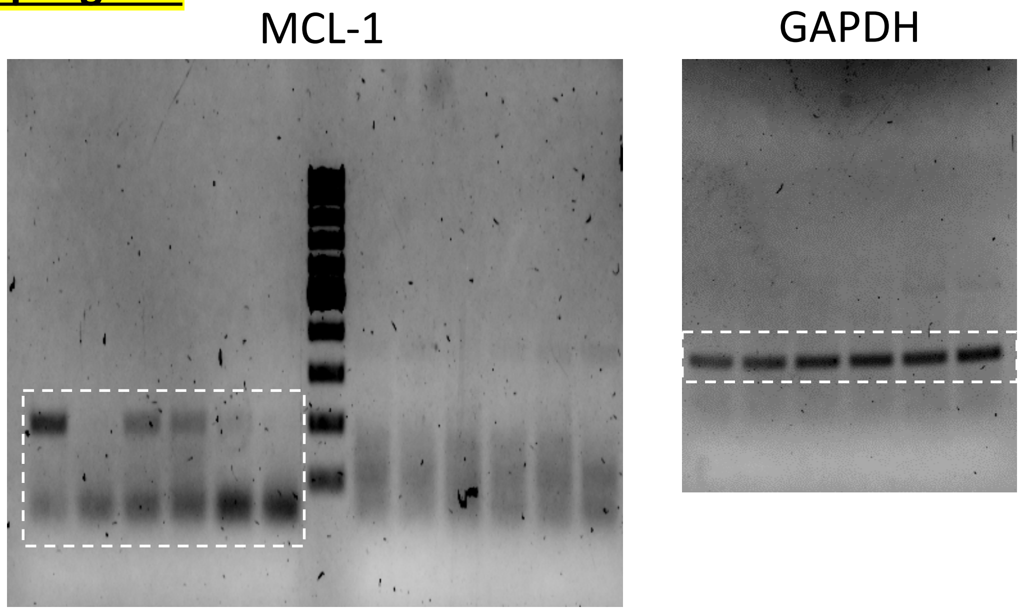

Sup Fig 13d

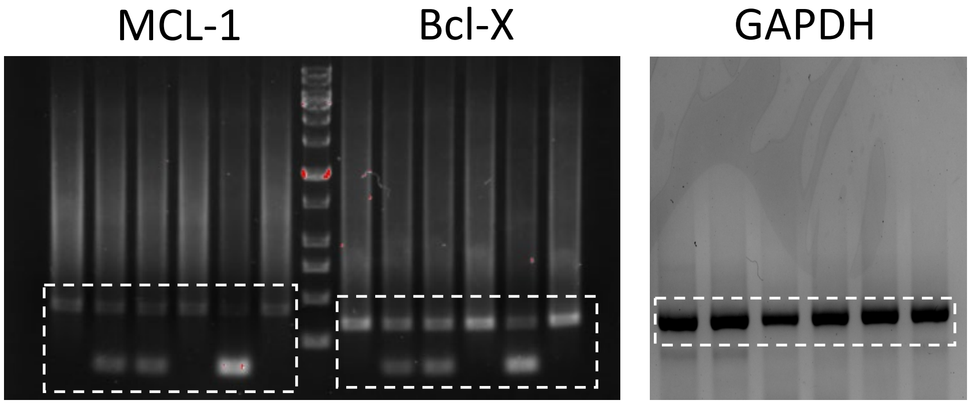

Sup Fig 14c

VEGF

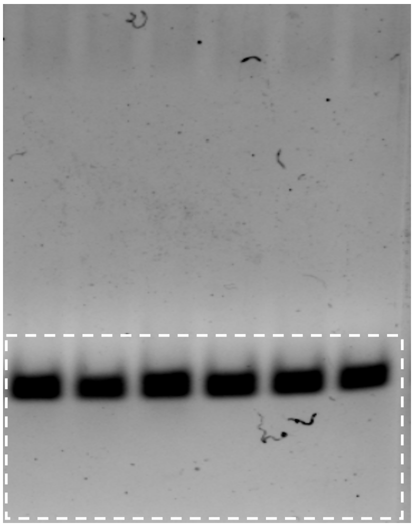

BAX

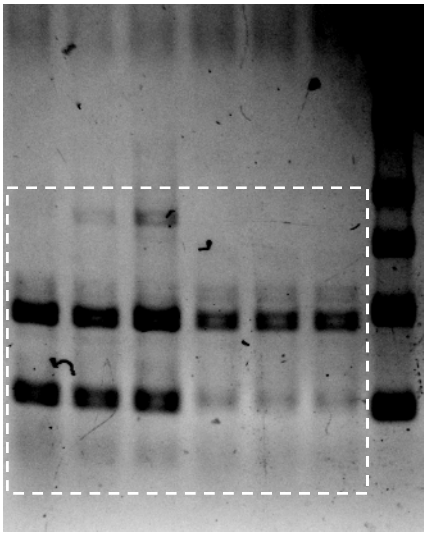

BARD1

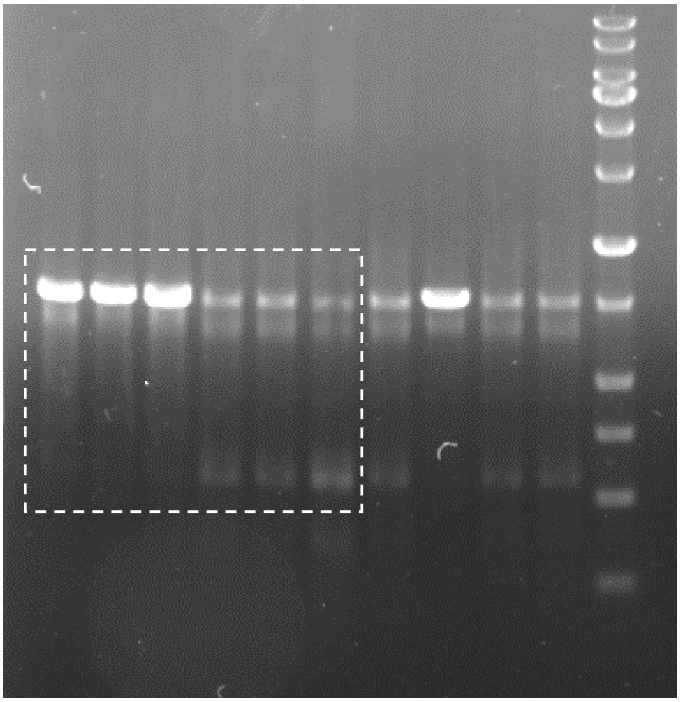

GAPDH

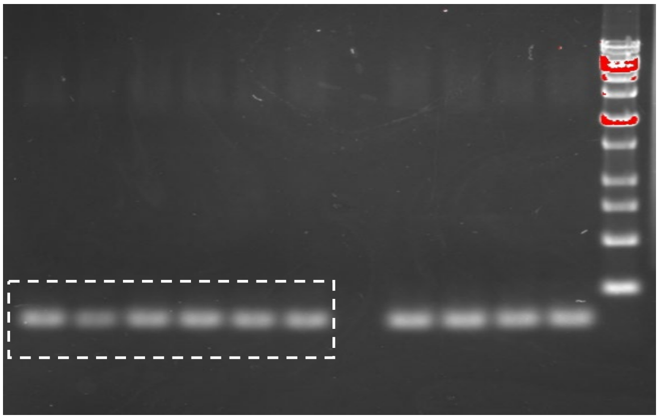

Sup Fig 14d

VEGF

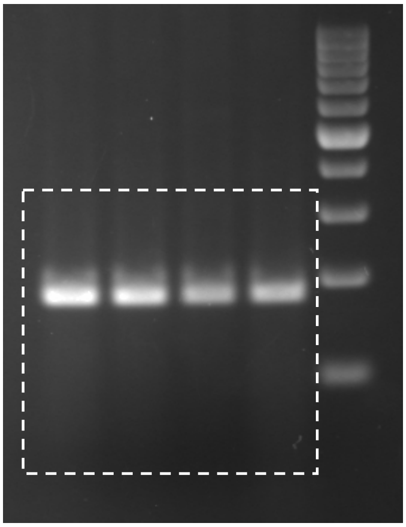

GAPDH

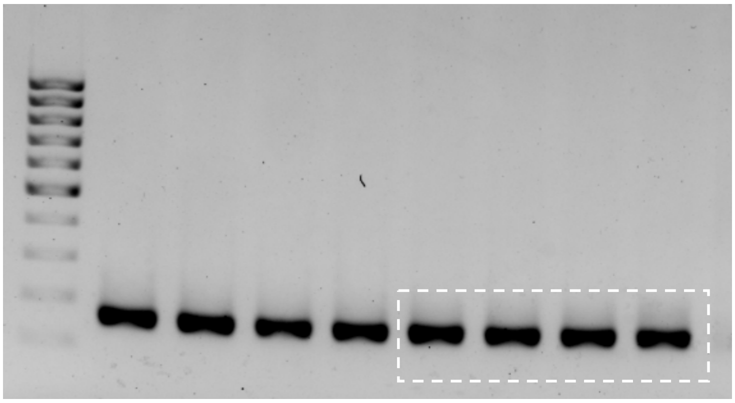

Sup Fig 14e

BAX

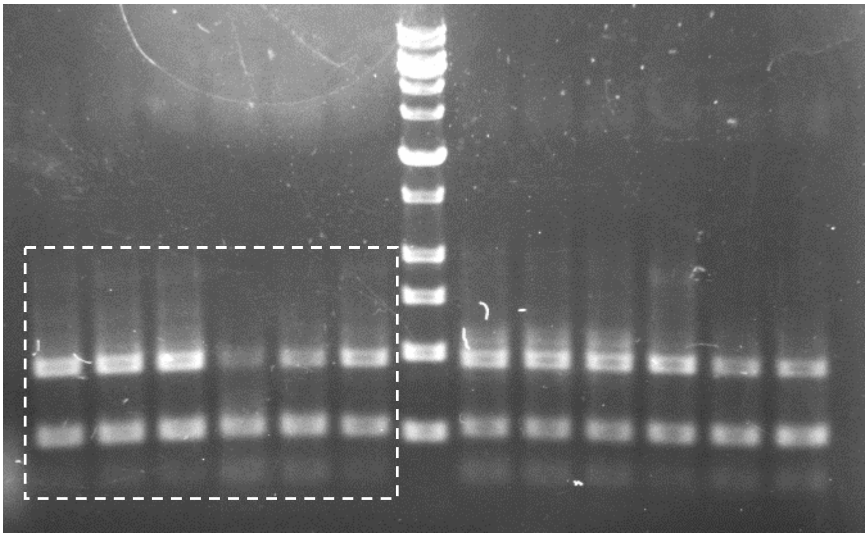

GAPDH

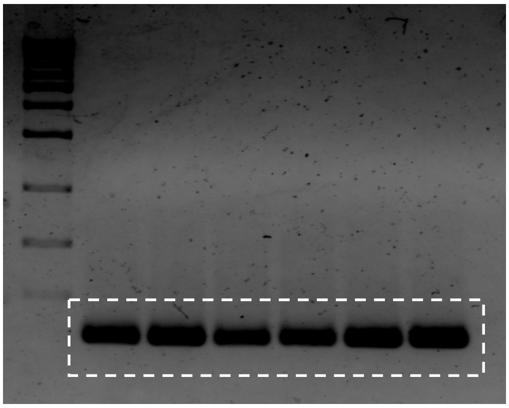

Sup Fig 15a

HCC70

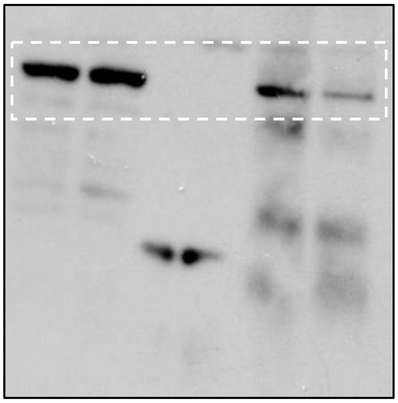

BT549

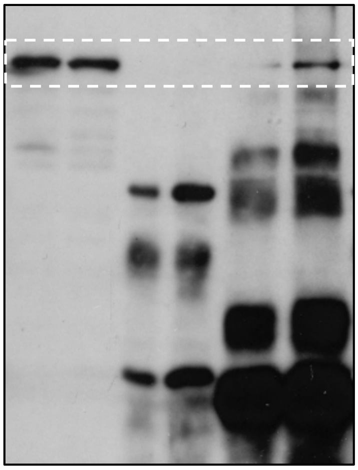

468

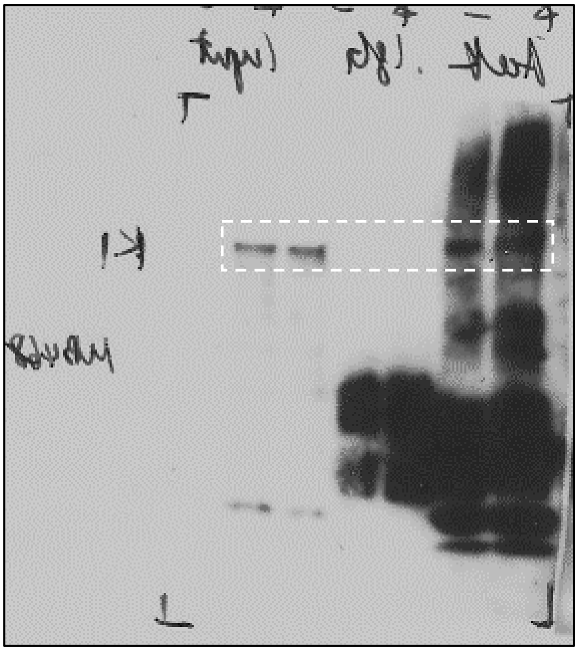

Sup Fig 15c

SRPK1

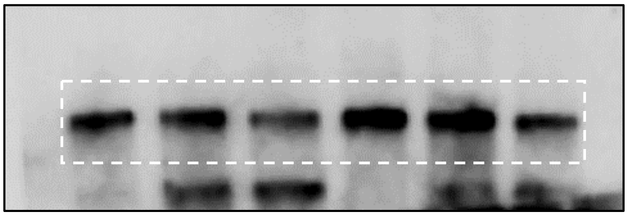

TIP60

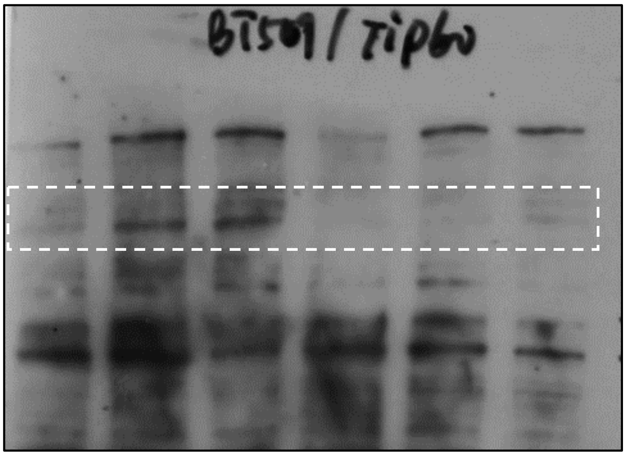

Actin

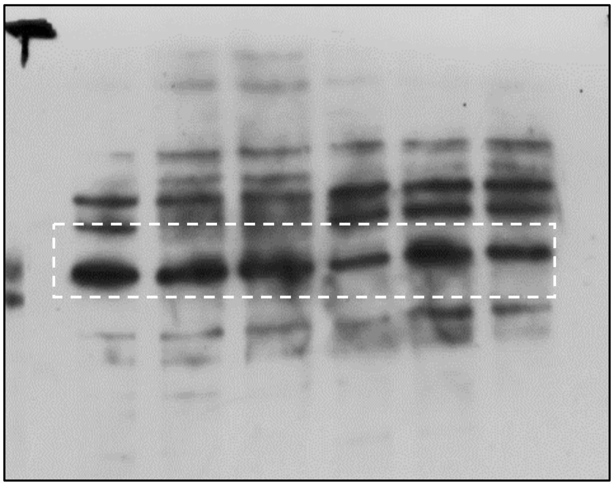

Sup Fig 15d

SRPK1

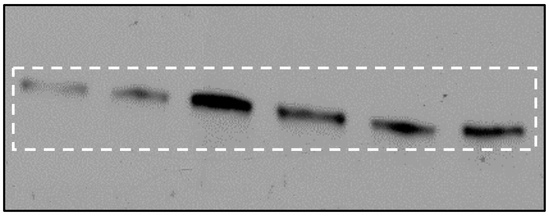

TIP60

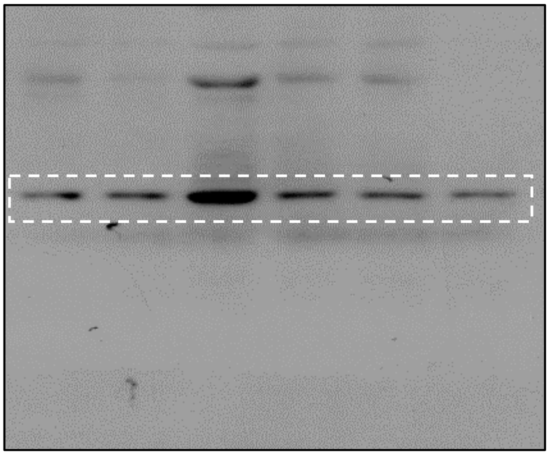

Actin

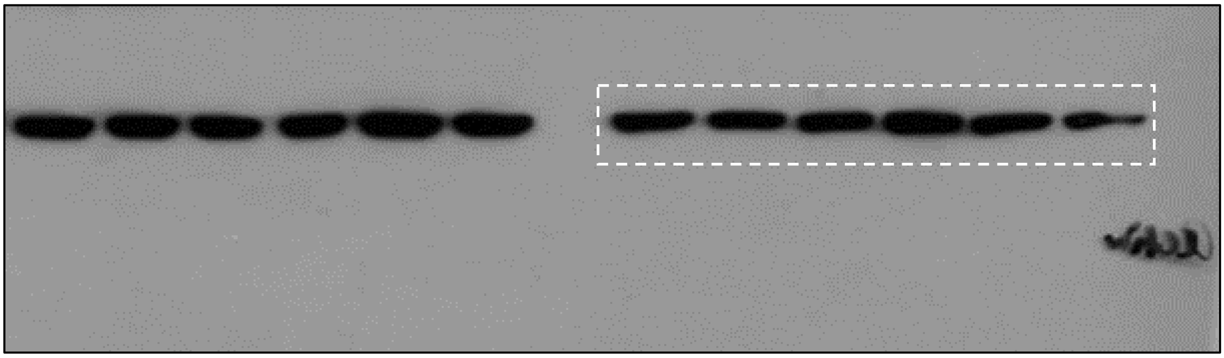

Sup Fig 15e

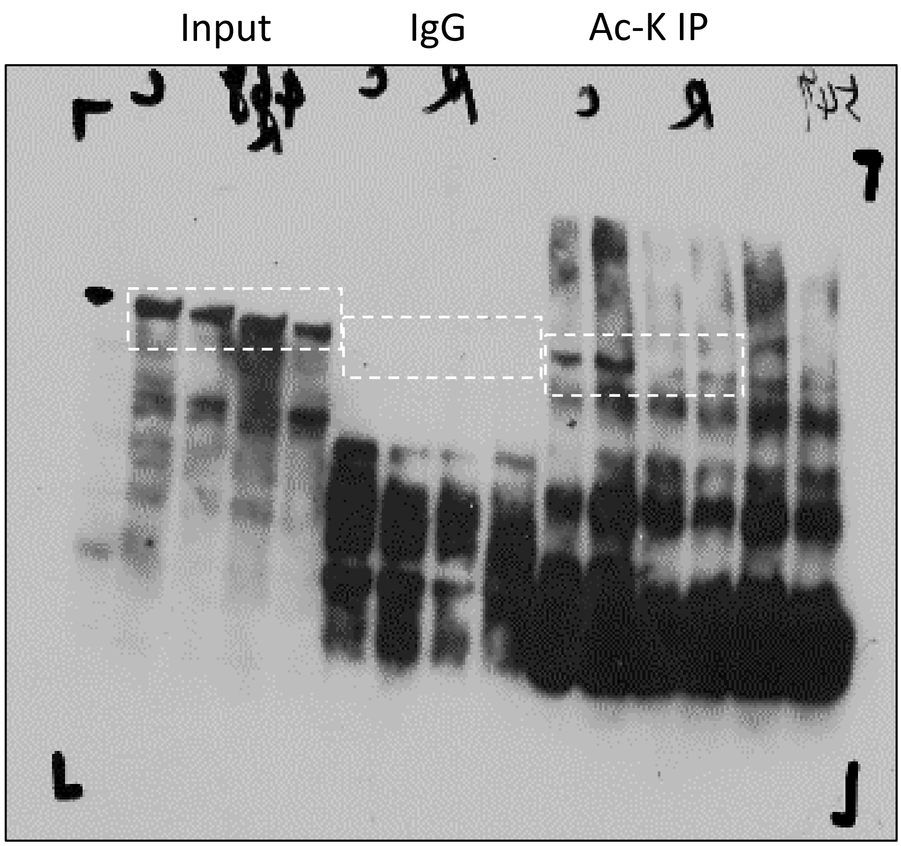

Sup Fig 15f

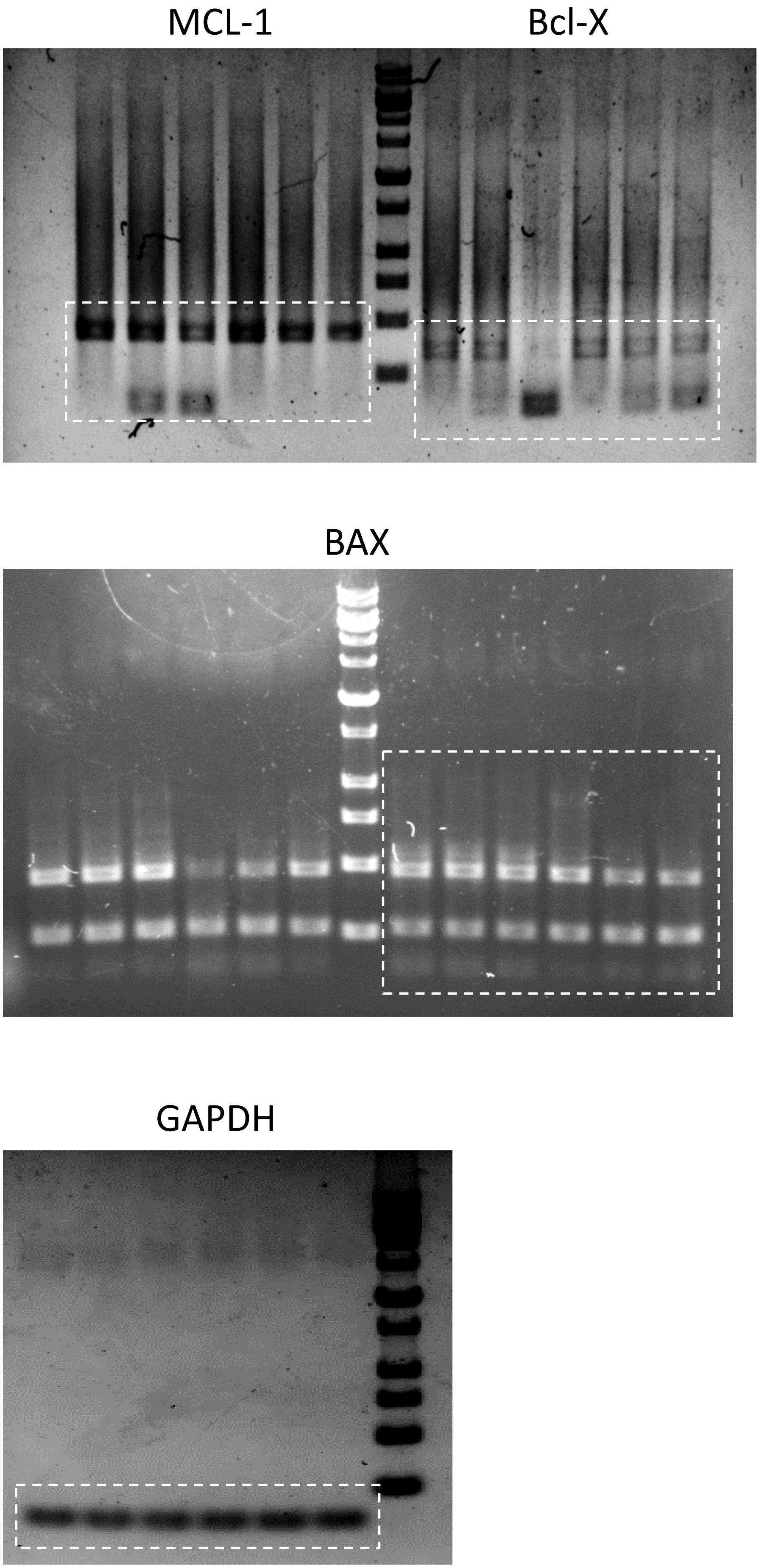

Sup Fig 17

Bcl-X

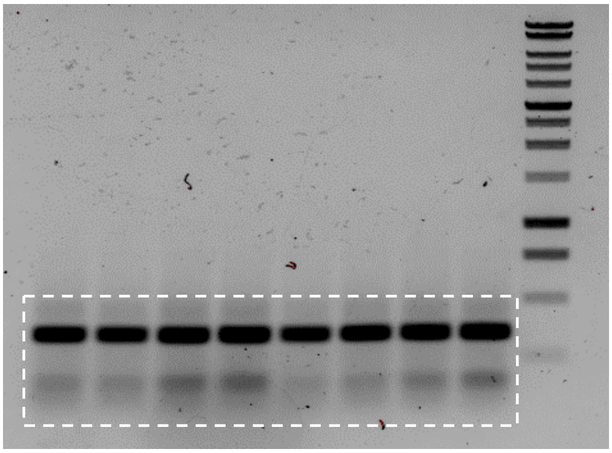

MCL-1

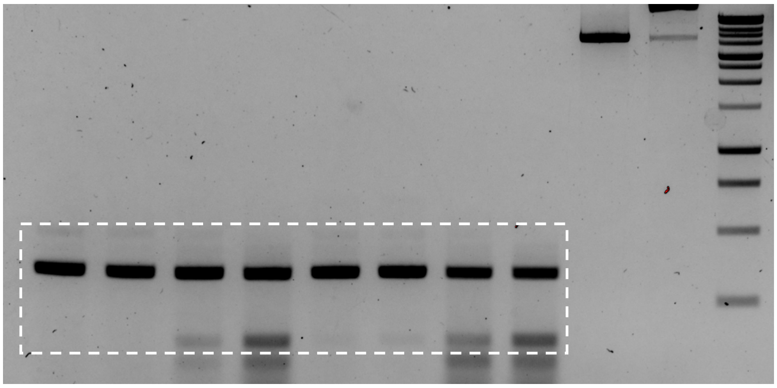

GAPDH

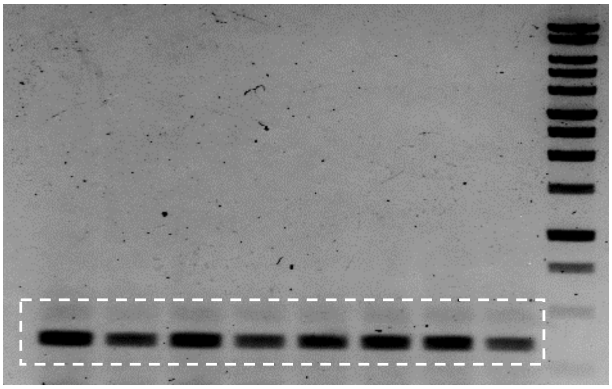

Supplement: Supplementary file 1 — Supplementary Information [file 42003_2020_983_MOESM1_ESM.pdf]
