# Supplementary material for: SRPK1 acetylation modulates alternative splicing to regulate cisplatin resistance in breast cancer cells
Source: Commun Biol. 2020 May 27;3:268. doi: 10.1038/s42003-020-0983-4 (PMC7253463; doi:10.1038/s42003-020-0983-4)
Supplement: Supplementary file 4 — Description of Additional Supplementary Files [file 42003_2020_983_MOESM4_ESM.pdf]

Description of Additional Supplementary Files

File Name: **Supplementary Data 1**
Description: This file includes the data presented in the bar graphs of the main figures.

File Name: **Supplementary Data 2**
Description: This file includes the potential post-translational modifications identified in SRPK1.
