# Supplementary material for: SRPK1 acetylation modulates alternative splicing to regulate cisplatin resistance in breast cancer cells
Source: Commun Biol. 2020 May 27;3:268. doi: 10.1038/s42003-020-0983-4 (PMC7253463; doi:10.1038/s42003-020-0983-4)
Supplement: Supplementary file 5 — Reporting Summary [file 42003_2020_983_MOESM5_ESM.pdf]

nature research

Corresponding author(s): Qidong Hu

Last updated by author(s): April 20, 2020

Reporting Summary

Nature Research wishes to improve the reproducibility of the work that we publish. This form provides structure for consistency and transparency in reporting. For further information on Nature Research policies, see Authors & Referees and the Editorial Policy Checklist.

Statistics

For all statistical analyses, confirm that the following items are present in the figure legend, table legend, main text, or Methods section.

n/a	Confirmed	
☐	☒	The exact sample size (*n*) for each experimental group/condition, given as a discrete number and unit of measurement
☐	☒	A statement on whether measurements were taken from distinct samples or whether the same sample was measured repeatedly
☐	☒	The statistical test(s) used AND whether they are one- or two-sided<br>*Only common tests should be described solely by name; describe more complex techniques in the Methods section.*
☒	☐	A description of all covariates tested
☒	☐	A description of any assumptions or corrections, such as tests of normality and adjustment for multiple comparisons
☐	☒	A full description of the statistical parameters including central tendency (e.g. means) or other basic estimates (e.g. regression coefficient) AND variation (e.g. standard deviation) or associated estimates of uncertainty (e.g. confidence intervals)
☐	☒	For null hypothesis testing, the test statistic (e.g. *F*, *t*, *r*) with confidence intervals, effect sizes, degrees of freedom and *P* value noted<br>*Give P values as exact values whenever suitable.*
☒	☐	For Bayesian analysis, information on the choice of priors and Markov chain Monte Carlo settings
☒	☐	For hierarchical and complex designs, identification of the appropriate level for tests and full reporting of outcomes
☒	☐	Estimates of effect sizes (e.g. Cohen's *d*, Pearson's *r*), indicating how they were calculated

Our web collection on statistics for biologists contains articles on many of the points above.

Software and code

Policy information about availability of computer code

Data collection	Microsoft Excel was used to collect the data
Data analysis	Microsoft Excel was used to analyze the data

For manuscripts utilizing custom algorithms or software that are central to the research but not yet described in published literature, software must be made available to editors/reviewers. We strongly encourage code deposition in a community repository (e.g. GitHub). See the Nature Research guidelines for submitting code & software for further information.

Data

Policy information about availability of data

All manuscripts must include a data availability statement. This statement should provide the following information, where applicable:

- Accession codes, unique identifiers, or web links for publicly available datasets
- A list of figures that have associated raw data
- A description of any restrictions on data availability

The statement is provided in the Method section. All the data are available upon request.

Field-specific reporting

Please select the one below that is the best fit for your research. If you are not sure, read the appropriate sections before making your selection.

☒ Life sciences ☐ Behavioural & social sciences ☐ Ecological, evolutionary & environmental sciences

For a reference copy of the document with all sections, see nature.com/documents/nr-reporting-summary-flat.pdf

Life sciences study design

All studies must disclose on these points even when the disclosure is negative.

Sample size	Sample size is at least three.
Data exclusions	No data were excluded from the experiments and analysis.
Replication	Most experiments in vitro and cell-based assays were repeated in at least three independent experiments, and the data were reproducible.
Randomization	For in vitro assays, cells were passaged equally among control and treatment groups. The allocation of cells for treatment was randomly assigned.
Blinding	Researchers were not blinded to samples.

Reporting for specific materials, systems and methods

We require information from authors about some types of materials, experimental systems and methods used in many studies. Here, indicate whether each material, system or method listed is relevant to your study. If you are not sure if a list item applies to your research, read the appropriate section before selecting a response.

Materials & experimental systems

n/a	Involved in the study	
☐	☒	Antibodies
☐	☒	Eukaryotic cell lines
☒	☐	Palaeontology
☒	☐	Animals and other organisms
☒	☐	Human research participants
☒	☐	Clinical data

Methods

n/a	Involved in the study	
☒	☐	ChIP-seq
☒	☐	Flow cytometry
☒	☐	MRI-based neuroimaging

Antibodies

Antibodies used

anti-SRPK1 (611072, BD Biosciences), anti-Hsp90 (610418, BD Biosciences), anti-Tip60 (DR1041, Calbiochem), anti-CLK1(ab74044, Abcam), anti-myc (#2276, Cell Signaling Tech.), anti-flag (#14793, Cell Signaling Tech.), anti-PARP (#9542, Cell Signaling Tech.), anti-phospho-H2AX (Ser139) (#9718, Cell Signaling Tech.), anti-β-actin(scc-47778, Santa Cruz), anti-GAPDH(#5174, Cell Signaling Tech.) and anti-lamin A(sc-6214, Santa Cruz)

Validation

SRPK1 (611072, BD Biosciences) Recommended for detecting human (QC Testing), mouse, rat and dog (Tested in Development) by IB and IHC/ICC; PMID: 8798720, PMID: 9472028
Hsp90 (610418, BD Biosciences) Recommended for detecting human Hsp90 (QC Testing), mouse, rat, dog and chicken (Tested in Development) by WB;
Tip60 (DR1041, Calbiochem) Recommended for detecting human Tip60 IB and IP; PMID: 16341205, PMID: 17189187
CLK1 (ab74044, Abcam); Recommended for detecting human CLK1 for IB, ICC and ELISA;
Myc (#2276, Cell Signaling Tech.) Recommended for detecting transfected myc-tagged proteins by IB, IP, IHC/ICC and FACS. PMID: 24366666

Flag (#14793, Cell Signaling Tech.); Recommended for detecting transfected flag tagged proteins by IB, IP, IHC/ICC and FACS. PMID: 30260624

PARP (#9542, Cell Signaling Tech.) Recommended for detecting endogenous full length and cleaved PARP1 resulting from Caspase cleavage in human, mouse, rat and monkey by IB. The antibody does not cross-react with related proteins or other PARP isoforms. PMID: 30577584

phospho-H2AX (Ser139) (#9718, Cell Signaling Tech.) Recommended for detecting endogenous levels of human, mouse, rat and monkey H2A.X only when phosphorylated at serine 139 by IB, ICC/IHC and FACS. PMID: 29978480

β-actin (scc-47778, Santa Cruz) Recommended for detecting β-actin from mouse, rat, human by IB, IP, IHC and FACS. PMID: 30602571

GAPDH (#5174, Cell Signaling Tech.) Recommended for detecting GAPDH from human, mouse, rat and monkey by IB and IHC/ICC. PMID: 30542722

lamin A(sc-6214, Santa Cruz) Recommended for detecting lamin-A from human, mouse and rat by IB, IF and ChIP. PMID: 29789551

Eukaryotic cell lines

Policy information about cell lines

Cell line source(s)	MCF-7 (HTB-22), MDA-MB-231(HTB-26), BT-549(HTB-22), MDA-MB-468(HTB-132) and MAb104 (ATCC® CRL-2067). All were obtained from ATCC.
Authentication	Cell lines have been authenticated by ATCC.
Mycoplasma contamination	Cell lines were tested for mycoplasma contamination by the vendor (ATCC). The investigators strictly adopted aseptic technique for cell culture
Commonly misidentified lines (See ICLAC register)	No misidentified cell lines were used.
